# From environmental burden to resource potential: environmental impact evaluation and Critical Raw Materials recovery from abandoned gold mining waste in the Western Alps (NW Italy)

**DOI:** 10.1007/s11356-026-38241-6

**Published:** 2026-09-26

**Authors:** Linda Zaniboni, Alessandro Cavallo, Domenico Antonio De Luca, Manuela Lasagna, Elio Padoan, Maria Martin, Giovanna Antonella Dino

**Affiliations:** 1 Department of Earth Sciences, University of Turin, Turin, Italy; 2 Department of Earth and Environmental Sciences, University of Milan-Bicocca, Milan, Italy; 3 Department of Agricultural, Forest and Food Sciences, University of Turin, Grugliasco, Italy

**Keywords:** Gold mines, Critical raw materials, Extractive waste characterization, Circular economy, Mining waste, Italy

## Abstract

Extractive waste (EW) from former mining activities poses long-term environmental impacts while representing a potential secondary resource. Within a circular economy framework, EW can be valorised to recover raw materials (RMs) and critical raw materials (CRMs) contributing to the supply of strategic resources. In the Anzasca Valley (Piedmont, Italy), gold mines of Crocette and Pestarena operated until the mid-20th century, producing large amount of EW. These materials have caused persistent contamination of soils and waters, particularly with As and other potentially toxic elements (PTEs). The aims of the study are i) to assess environmental contamination and ecological risk, and ii) to evaluate the potential for recovering CRMs from EW. To do so, the study combines mineralogical and geochemical data of soils and EW from different sampling campaigns. Results revealed significant enrichment of CRMs, particularly As, followed by W, Sb, Bi, and minor enrichment of some light REEs (La, Ce, Pr, Nd). The environmental impact assessment identified As as the main pollutant, classifying soils as heavily to extremely contaminated, with additional moderate contamination by Pb and Co, and indicating an overall moderate ecological risk. The estimated volumes of EW and residual amounts of selected CRMs (A, Bi, Sb, W) indicate a potential for concurrent recovery, with As as the primary target. The findings suggest that this approach can represent a sustainable intervention within remediation projects, combining environmental risk mitigation with the recovery of valuable resources and supply security.

**Supplementary Information:**

The online version contains supplementary material available at https://doi.org/10.1007/s11356-026-38241-6.

## Introduction

Mining activities legacy is often associate to the presence of abandoned mine sites, which are widespread all over the world (Venkateswarlu et al. 2016). These sites pose significant long-term risks to human health and the environment due to a lack of management, monitoring and rehabilitation (Candeias et al. 2019; Spitz and Trudinger 2019).

Mining activities represent an important source of waste production and, if not managed properly, can cause significant environmental impacts. At the European level, mining and quarrying represent the second largest source of waste generation, constituting 22.7% of the total waste by economic activities (Eurostat 2024), with almost 500 million tonnes of waste in 2022. The two main types of extractive waste (EW) generated from mining activities are waste rock (WR) and tailings (TA). Waste rock is defined as rock material excavated from underground workings or removed during preliminary sorting operations. It contains ore minerals at concentrations that are not economically recoverable but may nonetheless host significant amounts of raw materials (RMs) and critical raw materials (CRMs), often associated with the primary exploited ore mineral and either unrecognized or not utilized in the past. Tailings are defined as fine-grained waste materials produced during the mineral processing; these result from physico-chemical processes for the separation and concentration of ore minerals (BRGM 2001; Lottermoser 2010).

At abandoned mine sites, EW can be discarded in open air making it susceptible to erosion and leaching processes that impact the surrounding environment (Passariello et al. 2002; Mourinha et al. 2022). In the context of Au mines, EW typically contains sulfide minerals that can oxidise to produce acid mine drainage (AMD), or neutral mine drainage (NMD) when acid-neutralising minerals are present, which lead to the release of potentially toxic element (PTEs) (Ritcey 2005; Craw et al. 2015). Indeed, high concentrations of metals and metalloids in EW, such as As, Cd, Co, Cu, Ni, Pb, Zn, as well as ore processing chemicals such as cyanide and Hg can be mobilised in the environment (Barcelos et al. 2020; Laker 2023).

Contamination has been widely documented in soils surrounding former mining sites (Ferreira da Silva et al. 2004; Hou et al. 2023), in groundwater and surface water (Carvalho et al. 2014; Guerrieri et al. 2023), and across terrestrial and aquatic ecosystems, affecting microbial communities, vegetation, and bioindicators (Clark et al. 2021; Munford et al. 2023). These impacts may persist for decades after mine closure, posing ongoing risks to human health through direct and indirect exposure pathways (Coelho et al. 2007; Ngole-Jeme and Fantke 2017).

The social awareness on the environmental consequences of mining, as well as the growing demand for more sustainable practices to guarantee resources supply, fundamental for countries’ development (e.g. green and digital transition), is becoming increasingly relevant (Adiansyah et al. 2015; Aznar-Sánchez et al. 2018).

In the European context, Directive 2006/21/EC establishes specific obligations for safe and sustainable management of waste from extractive industries to minimise environmental and human health impacts (Garbarino et al. 2020). This approach is in line with the Circular Economy Action Plan (CEAP), which promotes sustainable production and the waste hierarchy (reduce, reuse, recycle, recover) to reduce pressure on natural resources (European Commission 2020). Based on this framework, the traditional 4R model has evolved into a 5R approach, in which waste is viewed as a resource and landfills are considered as anthropogenic deposits, enabling the recovery of valuable materials (Dino et al. 2018; Tayebi-Khorami et al. 2019; Kalisz et al. 2022).

In parallel, the increasing demand for CRM is addressed by the European Critical Raw Materials Act which emphasises the need to secure a sustainable and competitive supply of critical and strategic RM essential for clean energy and the EU’s climate and energy objectives (European Commission 2023a). In this context, EW represents a secondary source of RM, offering both economic and environmental benefits (Dino et al. 2018; Mancini et al. 2024; Machairas et al. 2025).

To minimise environmental risks and maximise the valorisation and recovery of EW, qualitative and quantitative characterisation is essential. This characterisation should be based on a multidisciplinary approach, collecting useful information to understand the composition, characteristics, and potential recovery of RM and/or CRM. To do so, it is necessary to undertake a comprehensive investigation into the history of the mining site, the type of the EW, and their volumes. Furthermore, a detailed waste characterisation through mineralogical and geochemical analyses is required (Amos et al. 2015; Lindsay et al. 2015).

The Anzasca Valley (Piedmont, NW Italy) hosts one of the main historical Au mining districts in the Western Alps. Abandoned EW facilities and associated EW discarded around processing plants have resulted in environmental contamination. At the same time, these EW deposits may constitute a valuable source of RM and CRM. The objective of this study is to evaluate whether and how WR and TA from the Anzasca Valley can be considered as secondary resources. Specifically, the research aims to: i) provide a qualitative and quantitative characterisation of WR and TA in terms of mineralogical and geochemical composition, extension, and volumes; ii) assess the current soil and sediment contamination and ecological risk; iii) explore EW potential for CRM recovery. The approach of treating EW as a RM resource can supports sustainable remediation, thereby mitigating environmental impacts.

## Study area

**Fig. 1:**
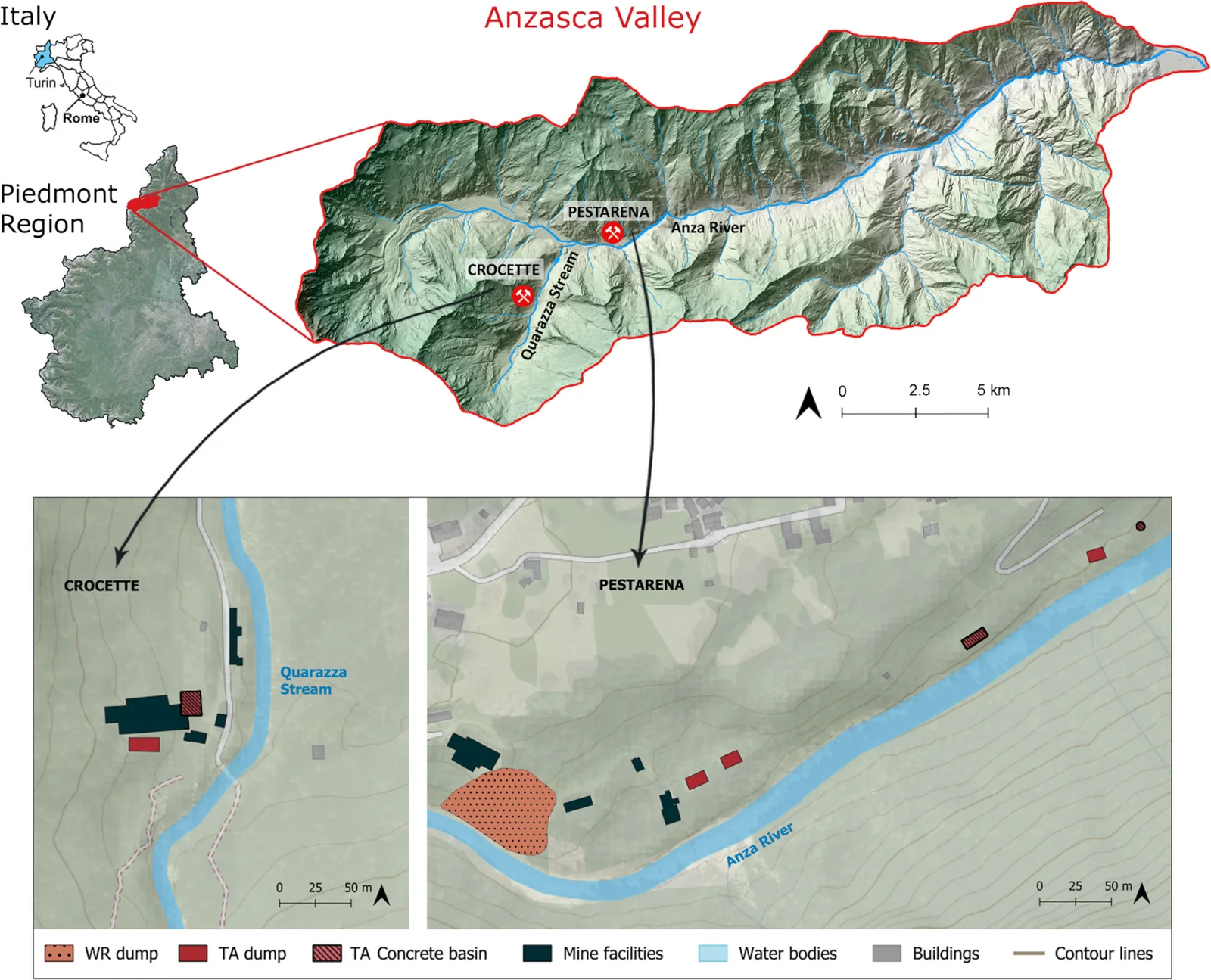
Geographical setting of the Pestarena and Crocette mines, with details of EW (WR dumps, TA dumps, TA concrete basins) and mining facilities

The present study focuses on two abandoned Au mines in the Anzasca Valley: Crocette and Pestarena (Fig. 1). The first official document attesting the presence of underground mining dates to the end of the 13^th^ century. The mine ceased operations in 1961 due to a decline in ore grade and rising operational costs (Bruck 1985; Cerri and Fantoni 2017). The ore bodies are hosted in paragneisses and micaschists in the Pestarena area, and in orthogneisses in the Crocette area. Mineralised veins are dominated by quartz and less abundant carbonates. Au is associated with sulphide minerals, mainly pyrite and arsenopyrite, with subordinate galena, sphalerite, pyrrhotite, and chalcopyrite (Lattanzi et al. 1989; Piana et al. 2017). The two main methods historically used in the Anzasca Valley for Au extraction were Hg amalgamation and cyanidation, introduced in the late 1880s (Bruck 1985; Cerri and Fantoni 2017).

Although no detailed records of historical waste management practices exist, some information can be reconstructed based on previous studies and field surveys. All these mining processes generated significant amounts of EW. Waste rocks were generally accumulated upstream of processing plants, while TA were accumulated downstream and in concrete basins near the respective facilities. Several years after mine closure, part of the TA were capped with HDPE geomembrane covers. However, it is believed that a considerable amount of EW was dispersed or abandoned in the surrounding areas close to the processing facilities (Fig. 1).

Previous studies have documented contamination linked to historical mining activities in the Anzasca Valley. Regarding the Crocette mining area, according to Allegretta et al. (2018), the soils and EW are heavily contaminated with As, with concentrations ranging from 145 to 40 220 mg/kg. The soils are characterised by low pH values ranging from 3.6 to 4.3. Integrated geochemical and mineralogical characterisation techniques revealed an absence of primary As-bearing minerals and the presence of amorphous Fe oxide-hydroxides, suggesting a high level of chemical weathering, as well as low leachability and bioavailability under stable conditions. However, a significant amount remains potentially mobilizable due to possible shifts in soil redox conditions and interactions with organic matter or biological activity.

The large scale study of Caviglia et al. (2015) investigated As concentrations in soil and surface water along the Quarazza stream, where Crocette is located, and downstream along the Anza River near Pestarena. They calculated, for these areas, the natural background value for soils, using different statistical methods. Applying the Upper Tolerance Limit (95 %) they found a value of 477 mg/kg As, much higher than the contamination threshold set by Italian legislation (20 – 50 mg/kg) (L.D. 152/2006). Moreover, many values found in soils near the mining areas were higher than the background by some orders of magnitude. Furthermore, surface water samples collected from the Quarazza Stream and the Anza River indicate that As concentrations in the most contaminated samples exceed the Italian limits for water (10 μg/L), reaching a maximum of 279 μg/L. The highest concentrations were found in areas close to mining facilities.

Zaniboni et al. (2025) analysed surface and groundwater quality at Crocette and Pestarena. The study revealed that 75% of the groundwater and surface water samples were contaminated with As (up to 70 μg/L). Although other PTEs remained at low concentrations in surface water, threshold values for Al, Mn, Fe, Ni and Pb were exceeded in Pestarena groundwater downstream of the TA, underscoring the persistent influence of EW.

In this context, the study also evaluates the current contamination of soil and sediment caused by mining activities, as well as the associated ecological risks.

## Materials and methods

The study combines data from unpublished works with results from a new sampling campaign. The previous studies (Bruno 2005; Nepote Valentin 2007; Rolando 2008) have investigated the Pestarena and Crocette mining area, collected and analysed samples of WR, TA, soil and sediments. Based on these datasets, we decided to conduct a new sampling campaign in 2024, focusing on areas where TA and WR seems to be present and expanding the investigation to include new areas. In total, 28 new samples were collected and mineralogical and geochemical analyses were performed on the new samples. Due to the complexity of the site and evidence of the dispersion and mixing of EW with surrounding soils, both earlier and newly collected samples were classified as WR, TA or soil under two alternative scenarios. This classification was based on geochemical and mineralogical results, as well as descriptions and information in the unpublished works and sample localisation. Based on the results, CRM abundance in EW was evaluated for recovery potential, and environmental impacts were assessed by applying contamination and ecological risk indices to soils and sediments.

### Previous samples collection and analyses

The study by Bruno (2005) included samples collected at Pestarena and Crocette from the 0–10 cm depth using a spatula, comprising soils, EW and sediments. At Pestarena, sediments were collected from both the drainage channel of the mine gallery and alluvial deposits of the Anza River, whereas at Crocette they were taken from a drainage channel discharging into the Quarazza River, likely draining the ore-processing area. Samples were air-dried, disaggregated, sieved at 2 mm, and quartered. A representative subsample was sieved at 0.5 mm, and the fraction retained between 2 mm and 0.5 mm was ground in an agate mortar. As, Cd, Co, Cr, Cu, Fe, Mn, Ni, Pb and Zn concentrations were determined following aqua regia digestion and analysis by Inductively Coupled Plasma–Atomic Emission Spectroscopy (ICP-AES). Mineralogical characterization was performed by X-ray diffraction (XRD) and Scanning Electron Microscopy coupled with Energy-Dispersive Spectroscopy (SEM-EDS).

Nepote Valentin (2007) investigated soils, EW and sediments from the drainage channel of the Pestarena mine gallery at Pestarena and Crocette. Samples were manually collected from the 0–15 cm depth, air-dried, disaggregated, sieved at 2 mm and quartered. A representative subsample was sieved at 0.5 mm and the fraction between 2 mm and 0.5 mm was ground in an agate mortar. Arsenic extraction was carried out using acid digestion according to EPA Method 3050B (HNO₃–H₂O₂), and concentrations were measured by ICP-AES. Mineralogical analyses of the fine fractions were conducted by XRD.

Rolando (2008) analyzed a sample of mineralized vein collected within a mine gallery at Pestarena. Prior to mineralogical investigation by XRD, samples were ground in an agate mortar to obtain very fine grains. For SEM-EDS analysis, the material was first cut using a circular saw and then prepared as a thin section approximately 25 μm thick. In addition, the study incorporated environmental characterization data from the Pestarena area produced by Regional Environmental Protection Agency (ARPA Piemonte), including borehole investigations and geochemical soil analyses aimed at quantifying PTEs and comparing their concentrations with the regulatory threshold values for contaminated sites established by Italian Legislative Decree 152/2006 (Sb, As, Be, Cd, Co, Cr, Cr(VI), Cu, Hg, Ni, Pb, Se, Sn, Tl, V, Zn, CN, F⁻).

The geochemical data produced by these previous studies were used in the present work to assess the environmental impact of the mining activities and to evaluate the potential for CRM recovery.

### New samples collection

A total of 28 new samples of WR, TA, soil and sediments were collected during two field campaigns in May and July 2024. Samples were taken using a hand shovel at 0-30 cm depth. The samples were then quartered to separate representative portions for analysis. The portions were then air-dried and milled (the coarser fraction having been previously crushed), then further quartered to obtain two subsamples: one for mineralogical analysis and one for geochemical analysis.

### Geochemical analyses

For geochemical analyses, the new samples were sent to Activation Laboratories Ltd. (Actlabs), an accredited laboratory where they were digested using a four-acid mixture (HF, HClO₄, HNO₃, and HCl). The resulting solutions were analysed by Inductively Coupled Plasma-Atomic Emission Spectroscopy ICP-AES for major elements, with additional trace elements determined by Inductively Coupled Plasma Mass Spectrometry (ICP-MS) for Ag, Al, As, Ba, Be, Bi, Cd, Ce, Co, Cr, Cs, Cu, Dy, Er, Eu, Fe, Ga, Gd, Ge, Hf, Ho, In, K, La, Li, Lu, Mg, Mn, Mo, Nb, Nd, Ni, P, Pb, Pr, Rb, Re, S, Sb, Sc, Se, Sm, Sn, Sr, Ta, Tb, Te, Th, Ti, Tl, Tm, U, V, W, Y, Yb, Zn, Zr.

### Mineralogical analyses

Mineralogical analyses on new samples were carried out using X-ray Powder Diffraction (XRPD) and SEM-EDS.

The XRPD analyses were performed using a PANalytical X’Pert PRO PW3040/60 X-ray diffractometer with Ni-filtered Cu Kα radiation at 40 kV and 40 mA, ½◦ divergence and receiving slits, and step scan of 0.02° 2θ, in the 3–80° 2θ range; the limit of detection of XRPD is approximately 0.5 % wt. The qualitative phase analysis was performed using the PANalytical HighScore Plus software version 2.2c using the ICSD PDF2-2008 database; quantitative phase analysis was carried out running the FULLPAT software (Chipera and Bish 2002). SEM-EDS analyses were carried out on carbon coated samples using a SEM (Vega TS Tescan 5163 XM) in combination with an EDS analyzer (EDAX Genesis 400), with 200 pA and 20 kV as standard conditions, using a series of minerals as standards (omphacite for Si and Ca; kaersutite for Al, K, Na and Ti; fayalite for Mg; spessartine for Fe and Mn; monazite for REE).

### Samples classification

To conduct a complete characterisation of the waste for environmental impact assessment and potential CRM recovery, the samples had to be distinguished (both new and previously collected) into the following categories: EW (i.e. TA and WR), soil, and sediments. Evidence from previous studies indicate that EW has dispersed throughout the area and progressively mixed with surrounding soils over time. Consequently, some samples could potentially fall into both the EW and soil categories. To account for uncertainty about past waste management and current condition, we hypothesised two scenarios:

- First scenario: a conservative approach, in which only samples that were clearly identified as TA or WR were classified as EW. These samples were considered EW as they were collected at, or in the immediate vicinity of, EW dumps. All other materials were classified as soil. In this scenario, the EW samples represent “indicated resources” when estimating recoverable CRMs.
- Second scenario: in addition to the EW identified in the first scenario, were also classified as TA or WR samples i) located in areas adjacent to the EW dumps; ii) with characteristics typically observed in the field, such as being in a vegetation-free area, having fine or coarse grain size (for TA and WR, respectively), or showing blue coloration indicative of the use of cyanide in the ore processing; iii) displaying high concentrations of metals and metalloids in geochemical analyses. All other samples were considered soil. In this scenario, the EW samples represent “inferred resources” in the assessment of recoverable CRMs.

**Fig. 2:**
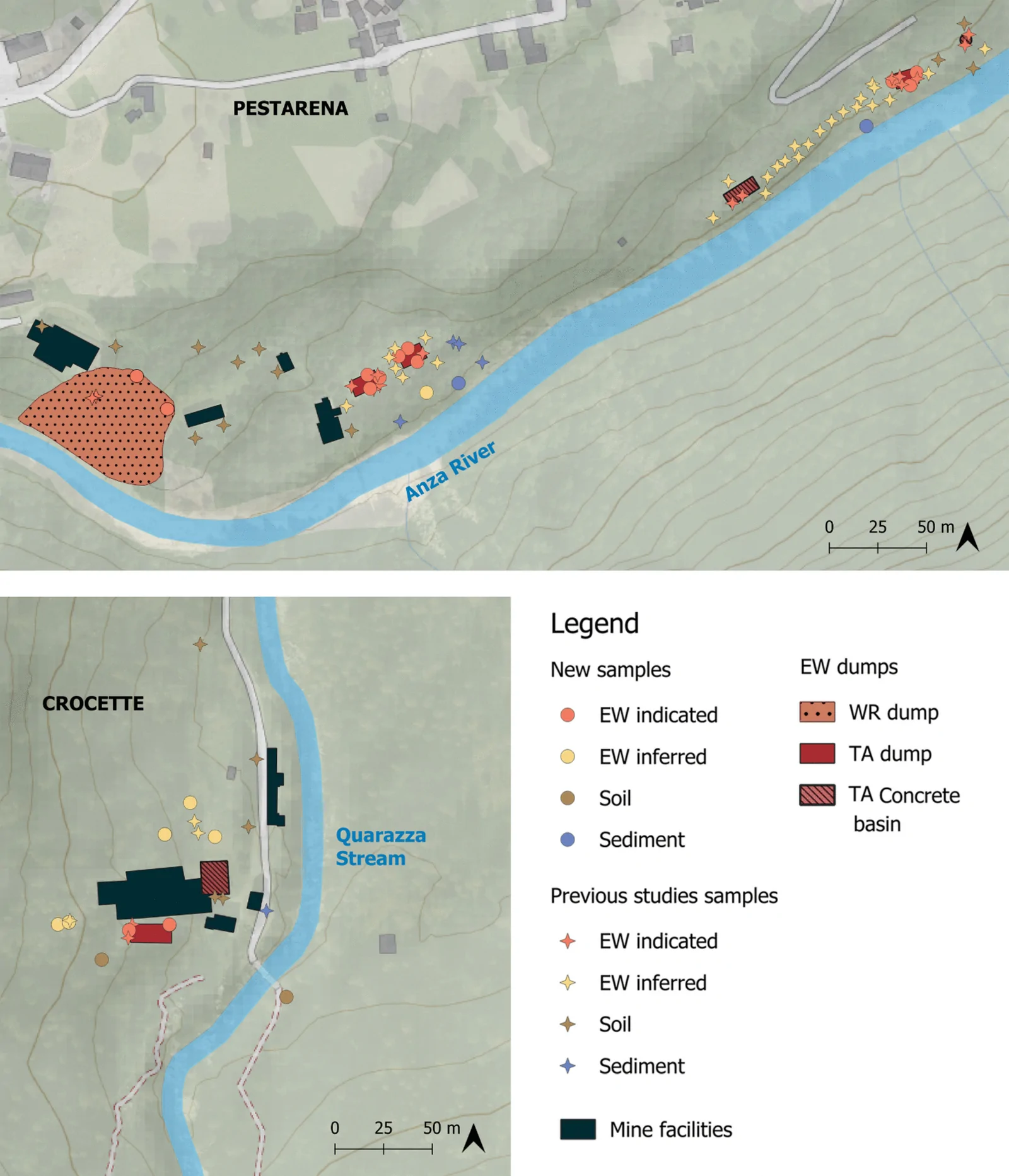
Sample location and classification. Circles indicate new samples (2024), stars indicate samples from previous studies. Colours represent sample types: orange = indicated EW, yellow = inferred EW (considered as soil in the first scenario and as EW in the second scenario), brown = soil, blue = sediment

Figure 2 shows sample location, categorisation and data source; additional information are reported in Supplementary Information (Table S1). As a complementary check, Principal Component Analysis and hierarchical clustering on multi-element compositions were also performed for both sites (Supplementary Information, Figs. S1–S2); the resulting statistical groupings did not correspond to the field-based classification, consistent with the extensive mixing of EW with surrounding soils discussed above.

### Data comparability

The dataset combines geochemical analyses from previous studies with those obtained from the new samples collected in 2024. The different sample collection, preparation and digestion procedures adopted across the datasets are described in the previous sections. These include involving different sample collection, preparation and digestion protocols: aqua regia digestion (Bruno 2005); EPA Method 3050B (HNO3–H2O2) for As only (Nepote Valentin 2007); official ARPA Piemonte analyses for regulatory site characterisation under Italian Legislative Decree 152/2006 (incorporated via Rolando 2008), for which the specific laboratory protocol is not reported in the available documentation, Italian regulatory practice for this type of characterisation is standardly based on aqua-regia acid digestion; and a four-acid (HF–HClO4–HNO3–HCl) digestion for the new 2024 samples.

To assess the comparability of the combined dataset, a Mann-Whitney U test was performed for each element measured in both the data from previous studies and the 2024 campaign, with a Benjamini-Hochberg correction for multiple comparisons (Table S2). No statistically significant difference was found between the two subsets for As, Cd, Cu, Ni, Pb, Sb and Zn (adjusted p > 0.05). This result supports the potential comparability of the two datasets for these elements, although it should not be interpreted as evidence of complete equivalence, given the differences in samples, spatial distributions, preparation and digestion procedures. Co, Cr and V showed a statistically significant difference (adjusted p < 0.05), with higher concentrations measured in the 2024 samples than in the historical subset. This pattern may partly reflect the greater effectiveness of four-acid digestion in recovering silicate-associated elements compared with aqua regia or EPA Method 3050B (US EPA 1996; Actlabs 2026). However, differences in sampling distribution and sample preparation may also have contributed to the observed pattern, and the relative contribution of analytical and spatial factors cannot be fully disentangled from the available dataset.

These results were used to guide the pooling of previous and new data in the subsequent analyses. For the environmental impact assessment, data were used for As, Cd, Co, Cr, Cu, Ni, Pb and Zn; results are reported as distributions and ranges rather than single point estimates, preserving the variability associated with the combined dataset. For Co and Cr, the higher concentrations observed in the 2024 samples may indicate that the historical data provide lower measured concentrations under the analytical and sampling conditions used. Accordingly, the resulting Igeo, CF and ERI/PERI values for these two elements are conservative, and their true contribution to ecological risk may be marginally higher than reported. The same approach was adopted for the CRM recovery potential assessment, where previous and new data were pooled for As, Co, Cu, Sb and V. For As, Cu and Sb, the absence of statistically significant differences between the subsets supports their combined use. For Co and V, the higher concentrations observed in the 2024 samples may result in lower UCC-normalised enrichment estimates when the previous data are included. However, this potential effect should be interpreted cautiously, as the observed differences may reflect both analytical and sampling conditions. The reported enrichment values for these elements should therefore be regarded as potentially conservative estimates, while acknowledging that their actual enrichment in the EW may be higher than indicated by the combined dataset.

### Environmental impacts

Contamination was assessed using the following indices for both soils and sediments (Hou et al. 2023; Di Duca et al. 2024; Dantas et al. 2025): geo-accumulation index (Igeo), contamination factor (CF), ecological risk index (ERI) and corresponding potential ecological risk index (PERI).

The geo-accumulation Index (Igeo) used to evaluate soil and sediments contamination using as reference the background values, to determine whether these pollutants are of natural or anthropogenic origin. It is calculated using the Eq. 1 (Muller 1969).

1 $${I}_{geo}={Log}_{2}\left[{C}_{i}/1.5\times {B}_{i}\right]$$

The contamination factor (CF) is an index used to evaluate the degree of pollution in soils and sediments for a single contaminant. Combining the CF with the toxicity factor of each element results in the ecological risk index (ERI). The sum of the ERI values for different contaminants yields the potential ecological risk index (PERI), accounting for the combined effects of multiple PTEs. CF, ERI, and PERI are calculated using the Eqs. 2–4 (Hakanson 1980).

2 $${CF}_{i}={C}_{i}/{B}_{i}$$

3 $${ERI}_{i}={T}_{r}^{i}\times {CF}_{i}$$

4 $$PERI=\sum_{i}^{n}{ERI}_{i}$$

where *CF*_*i*_ is contamination factor of the* i* PTE; ${T}_{r}^{i}$ is the toxic-response coefficient of the PTE (As = 10, Cu = 5, Cd = 30, Ni = 5, Pb = 5, Cr = 2, Zn = 1, Mn = 5).

ERI < 5 low risk; 5 ≤ ERI < 10 moderate risk; 10 ≤ ERI < 20 significant risk; 20 ≤ ERI < 40 high risk; ERI ≥ 40 very high risk.

PERI ≤ 150 low risk; 150 < PERI < 300 moderate risk; 300 < PERI < 600 high risk; PERI ≥ 600 significantly high risk.

### Potential recovery

The concentrations of CRMs listed by the European Union (European Commission 2023a) in previous and new samples were normalised to upper continental crust (UCC) values (Rudnick and Gao 2014) to identify elements enriched in EW that are suitable for recovery.

The following Eq. 5 and Eq. 6 were used to estimate the quantity of selected residue CRMs in EW under the two scenarios:

5 $${\mathrm{Q}}_{\mathrm{I}} = {V}_{\mathrm{I}} \times \uprho \times \mathrm{c}$$

6 $${\mathrm{Q}}_{\mathrm{I}\mathrm{I}}= {\mathrm{V}}_{\mathrm{I}\mathrm{I}}\times \uprho \times \mathrm{c}$$

where *Q*_*I*_ is the residual quantity of the considered CRM in the first scenario, where indicated resources correspond to EW dumps; *Q*_*II*_ is the residual quantity of the considered CRM in the second scenario, where inferred resources also include a wider area assumed to potentially be EW; *V*_*I*_ is the volume of indicated resources; *V*_*II*_ is the volume of indicated resources; *ρ* is the bulk density of EW (1.5 g/cm^3^ for WR and 1.8 g/cm^3^ for TA (Dino et al. 2018); c is the average concentration of the considered CRM from geochemical analyses.

To assess the theoretical economic value of EW the following Eq. 7 and Eq. 8 were used:

7 $${EV}_{I}= {\mathrm{Q}}_{\mathrm{I}} \times \mathrm{R} \times \mathrm{m}\mathrm{v}$$

8 $${EV}_{II}= {\mathrm{Q}}_{\mathrm{I}\mathrm{I}} \times \mathrm{R}\times \mathrm{m}\mathrm{v}$$

where *EV*_*I*_ is the economic value under scenario I; *EV*_*II*_ is the economic value under scenario II; *R* is the recovery rate; *mv* is the market value (in €) of the considered CRM, based on the average price reported for 2000–2021 (European Commission 2023b).

C_i_ is the concentration of the *i* PTE measured in the soil sample (mg/kg), *Bi* is the background value of the *i* PTE (mg/kg). The *Bi* values for Co, Cr, Cu, Ni, Pb, Zn refer to the natural background values for soils as calculated for homogeneous concentration areas in the Piedmont region (ARPA Piemonte 2019). The *Bi* value for As refers to the background value identified in the mining areas (Caviglia et al. 2015).

Igeo ≤ 0 Practically uncontaminated; 0 < Igeo ≤ 1 uncontaminated to moderately contaminated; 1 < Igeo ≤ 2 moderately contaminated; 2 < Igeo ≤ 3 moderately to heavily contaminated; 3 < Igeo ≤ 4 heavily contaminated; 4 < Igeo ≤ 5 heavily to extremely contaminated; Igeo > 5 extremely contaminated.

## Results and discussion

### Mineralogical analyses

The mineralogy of the mineralized veins provides critical insights into the original conditions of the ore minerals prior to any processing. In the Au-bearing vein sample, the main minerals were arsenopyrite, pyrite, quartz, rutile, sphalerite and covellite, which potentially resulted from the alteration of chalcopyrite.

The mineralogical composition of EW samples collected from the Crocette and Pestarena mining areas comprised mainly quartz, feldspars (both plagioclase and K-feldspar), and micas (muscovite, biotite, and/or illite); chlorite is frequently present as well, formed by the hydrothermal alteration of biotite. This mineralogical composition reflects that of the host rocks and mineralised veins, consistent with the literature for the Anzasca Valley (Curti 1987; Lattanzi et al. 1989).

Secondary minerals identified include: (I) Scorodite: originates from the alteration of As-bearing primary minerals such as arsenopyrite (Craw and Chappell 2000; Assawincharoenkij et al. 2018). (II) Jarosite: forms under high concentrations of Fe and SO₄^2^⁻ through oxidation processes involving pyrite and pyrrhotite (Sracek et al. 2004; Lindsay et al. 2015). (III) Alunite: a secondary mineral formed through the alteration of aluminosilicates (Amos et al. 2015; Jamieson et al. 2015).

Minor minerals include amphiboles, zeolites, rutile, garnet, dolomite, scheelite, and other clay minerals. Scheelite, a calcium tungstate, is one of the main W ores and among the easiest to dissolve (Li et al. 2017)

**Fig. 3:**
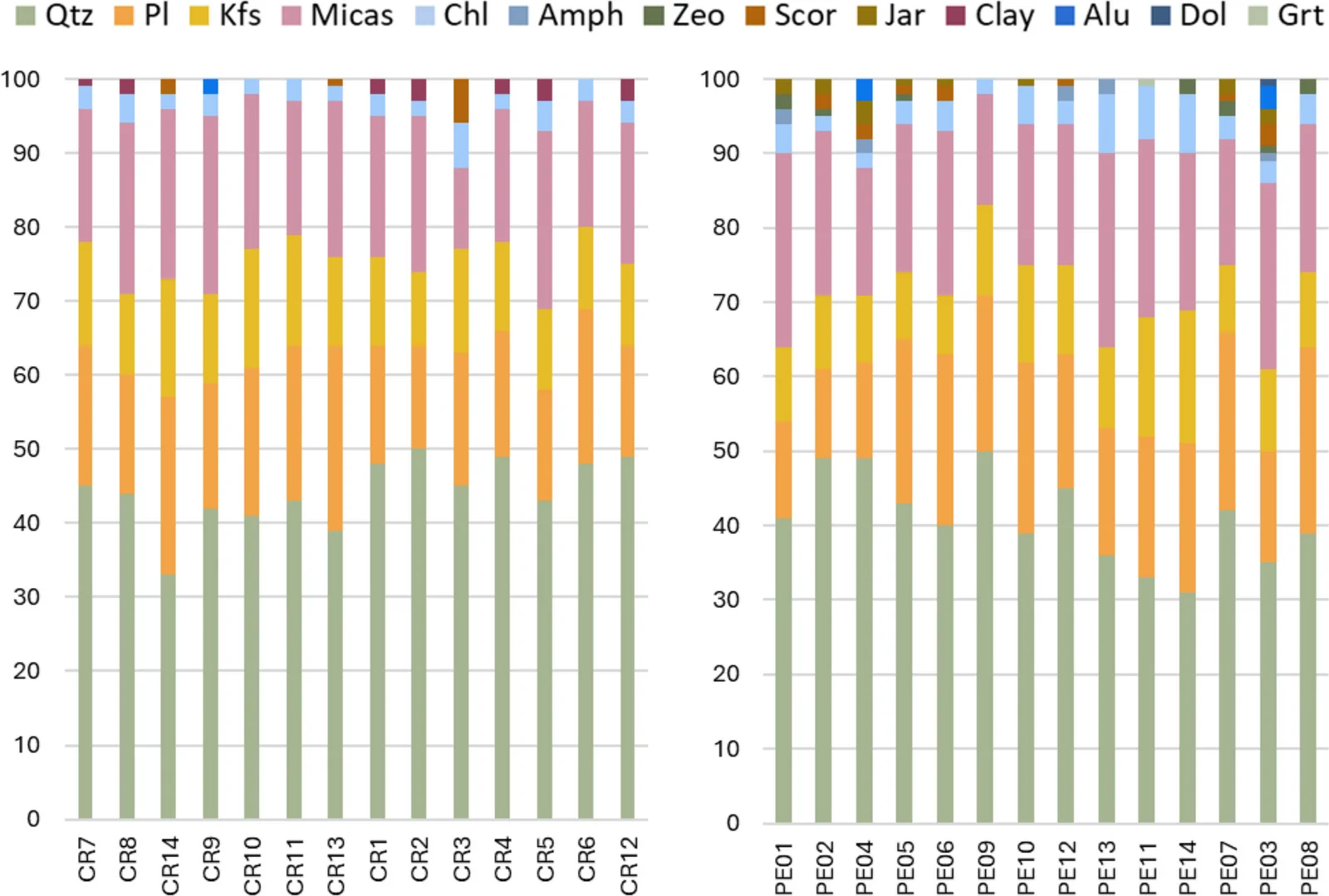
Main minerals in the new samples from Crocette (CR) and Pestarena (PE): Qtz = quartz; Pl = plagioclase (abite); Kfs = K-feldspar; Micas = muscovite and biotite (and/or illite); Chl = chlorite; Amph = amphibole (mainly hornblende and/or tremolite); Zeo = zeolites; Scor = scorodite; Jar = jarosite; Clay = clay minerals (other than illite and chlorite); Alu = alunite; Dol = dolomite; Grt = garnet

Detailed mineralogical results for the newly collected samples are shown in Fig. 3. At Crocette, the inferred coarse WR (CR3, CR9) show the presence of scorodite, jarosite, and alunite indicative of sulphide oxidation processes. The inferred fine WR (CR1, CR2, CR4, CR5) are richer in micas and clays. In the TA (CR7, CR8, CR10, CR11, CR13, CR14), secondary minerals alunite, jarosite and scorodite are more frequently observed, although often only in trace amounts. In general, no significant mineralogical differences emerge among the samples. Similarly, at Pestarena, no clear mineralogical differences are observed among the samples. The TA (PE01, PE02, PE04, PE05, PE06, PE09, PE10, PE12, PE13) have a similar composition, with high contents of quartz and feldspars, abundant micas, and occasional occurrences of alteration secondary minerals such as alunite, jarosite, and traces of scorodite. The WR (PE11, PE14), in contrast, are less altered and lack these phases. Among the sediments, those from the drainage of the mine tunnel (PE03) show evidence of alteration, with the presence of alunite, jarosite, and scorodite.

SEM-EDS analyses also revealed (Fig. S3): (I) Monazite-(Ce), a REE-bearing phosphate mineral, typically containing up to 70% REE oxides, in granules ranging in size from approximately 60 to 180 µm. Its composition is dominated by Ce, with significant amounts of La, Pr and Nd (Jordens et al. 2013). (II) Traces of residual arsenopyrite and pyrite. (III) In some cases, traces of ilmenite. Ilmenite and rutile are the main minerals from which titanium is produced (Force 1991). (IV) Traces of zircon and barite.

### Geochemical analyses

The concentrations of the identified CRMs (as listed by the European Union) in the samples were normalised to the UCC values (Rudnick and Gao 2014). Normalization helps to identify which elements are significantly enriched or depleted in EW (Singh et al. 2011; Dai et al. 2016). This analysis was conducted under two previously defined scenarios: the first scenario considered the indicated EW only, while the second scenario also included the inferred EW.

**Fig. 4:**
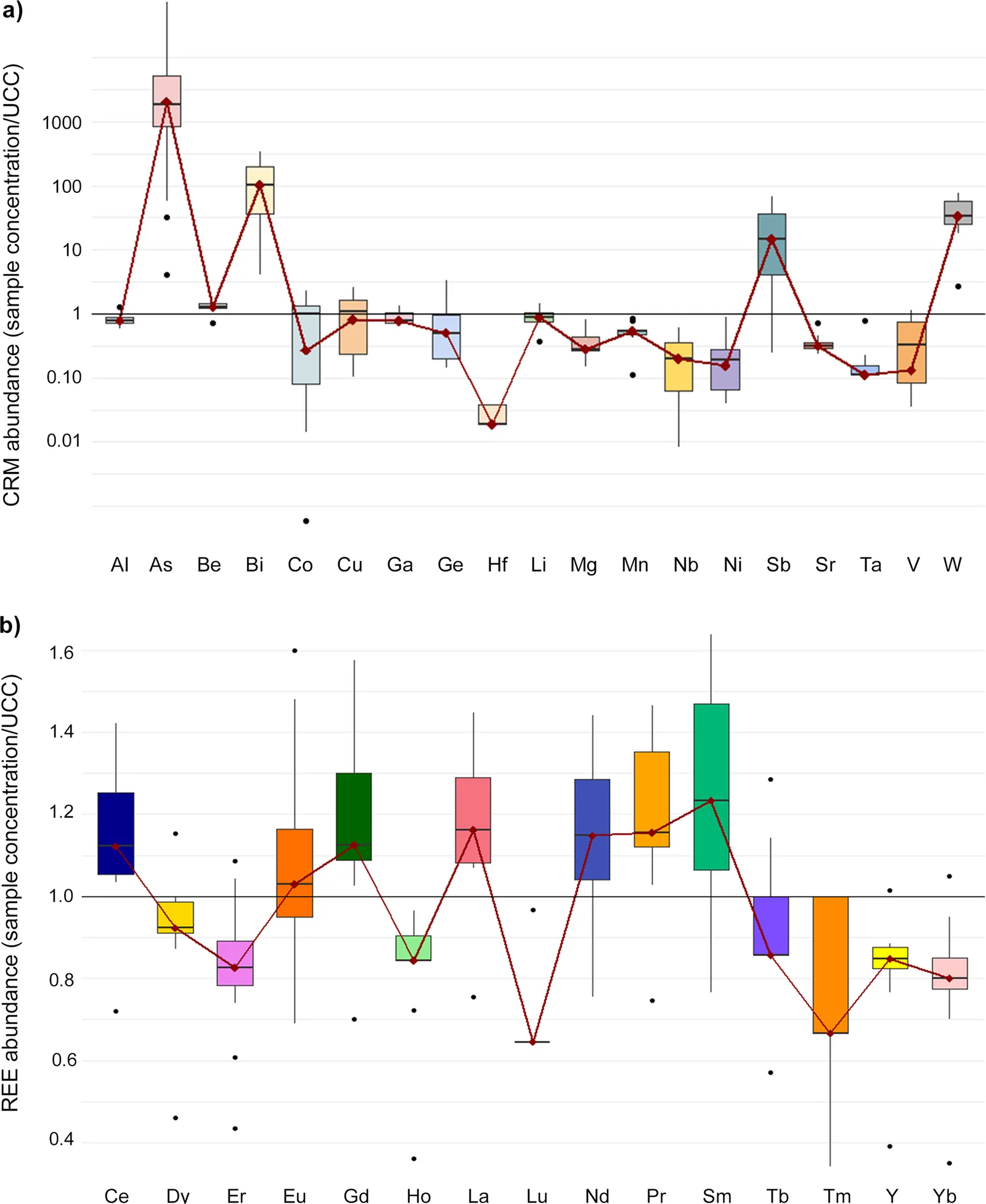
Abundance of CRMs (**a**) and REEs (**b**) at the Pestarena site. The first scenario is represented by boxplots showing the distribution of normalized concentrations (concentration/UCC) for each element, and the second scenario by red markers indicating the normalized median values

At Pestarena (Fig. 4a), As shows high enrichment, with a median values 1700 times higher than the UCC in the first scenario and up to 2000 times higher in the second scenario. For both scenarios Bi, W and Sb also exhibit enrichment, with median values of about 100, 35 and 15 respectively. Be exhibits slight enrichment with median value of 1.3. In some samples Cu, Co, Ga, and Ge in values exceed 1 but the median values are close to the UCC, while the values for the other elements are below 1.

For what concerns REEs abundance (Fig. 4b), Tm, Yb, Lu, Y, Ho, Er, Tb, and Dy show values below 1, while Eu is close to 1. La, Ce, Pr, Nd, Sm, and Gd show slight enrichment, with median values ranging from 1.14 to 1.26 in both scenarios.

**Fig. 5:**
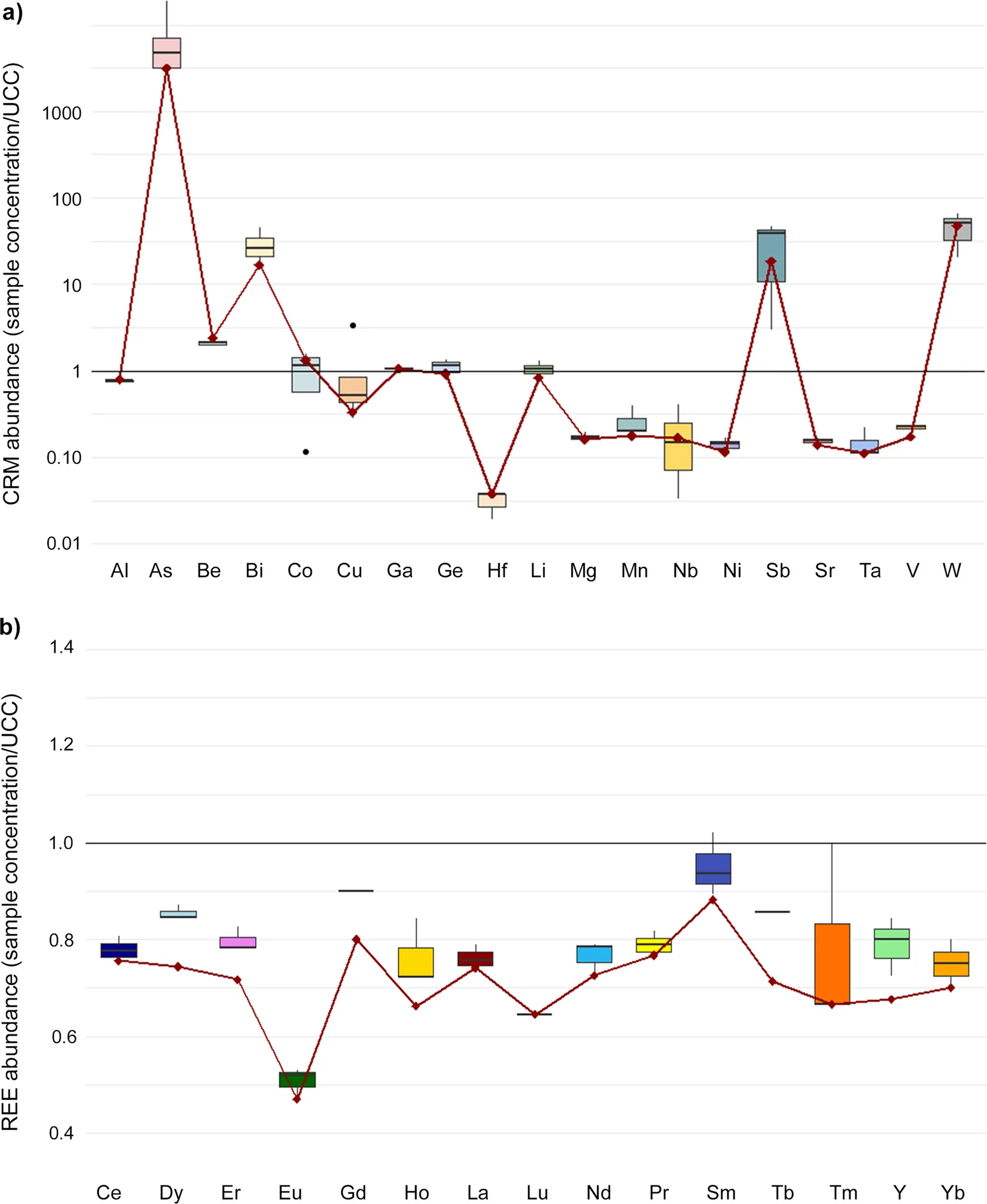
Abundance of CRMs (**a**) and REEs (**b**) at the Crocette site. The first scenario is represented by boxplots showing the distribution of normalized concentrations (concentration/UCC) for each element, and the second scenario by red markers indicating the normalized median values

At Crocette, under the first scenario, enrichment is observed for some CRMs. Arsenic shows the highest enrichment, with a median value around 4000 times higher than the UCC. This is followed by median enrichment values of 50 for W, 40 for Sb and 26 for Bi. Be exhibited slight enrichment (median 2), whereas Al, Co, Ga, Ge and Li show values close to 1, indicating no significant enrichment. Other analysed elements have values below 1. Under the second scenario, the overall trend remains consistent, with a slight decrease in median enrichment values for As, Bi, Sb, and W, and a minor increases in Be concentrations (Fig. 5a). The REEs are generally not enriched in either scenario, with mean values below or near 1 (Fig. 5b).

### Environmental impacts

Environmental impacts were assessed on sediments and soils under the two scenarios. In the first scenario, only the samples collected within the EW dumps were used (*indicated*), while in the second scenario, a larger number of samples were classified as EW (*inferred*), thereby expanding the area considered. Only the outermost samples, which were slightly more distant from the dumps, were categorised as soil.

#### Sediment

The river sediments were found to be uncontaminated and not to represent a potential ecological risk (Igeo ≤ 0; PERI ≤ 150). Instead, the large oxidisable surface area exposed by mining excavation in the tunnels, as well as leaching from EW results in ecological risk in sediments samples collected at the drainage of the mine tunnels in Pestarena and at the drainage channel flowing out from the TA dumps in Crocette (Table S3). For these samples at Pestarena Igeo values indicate heavily to extremely As contamination, moderately to heavily Pb contamination, and moderate Co and Zn contamination. The PERI values indicate a high ecological risk, with the main contribution from As, that shows very high risk, followed by a high risk associated with Pb and Cu, and a significant risk associated with Co. At Crocette, Igeo values indicate heavy As contamination. Moderate PERI values reflect high risk for Pb and very high risk for As. PTEs associated with the contaminated sediments, which represent potential ecological risk, may be subject to changes in mobility and availability in response to variations in hydrological and geochemical conditions (Sanchez et al. 2025). During low-flow periods, the oxidation of sulphide-bearing materials by oxygenated water promotes the release of PTEs, while the formation of secondary Fe (hydr)oxides act as a contaminant sink through adsorption and co-precipitation (Bidone et al. 2016; Craw and Pope 2017; Gomes and Valente 2024). During periods of increased discharge, high-flow events may promote the erosion and resuspension of these sediments, enhancing the downstream transport of particle-bound PTEs (Dedieu et al. 2014; Zhang et al. 2014). In sediments, local redox condition can also influence PTEs mobility, as prolonged water saturation and organic matter degradation promote reducing conditions, the reductive dissolution of Fe (hydr)oxides may release previously sequestered PTEs into the water column, while dissolved organic matter may further affect their mobility and transport (Bauer and Blodau 2006; Valente et al. 2011; Yang et al. 2015; Santos et al. 2023). Downstream of the drainage PTE concentrations are expected to progressively decrease through dilution of uncontaminated waters (Larkins et al. 2018). Therefore, the contaminated sediments may act as a long-term sink for PTEs, contributing to the persistence of contamination, while also representing a potential secondary source of PTEs to downstream waters.

#### Soil

**Fig. 6:**
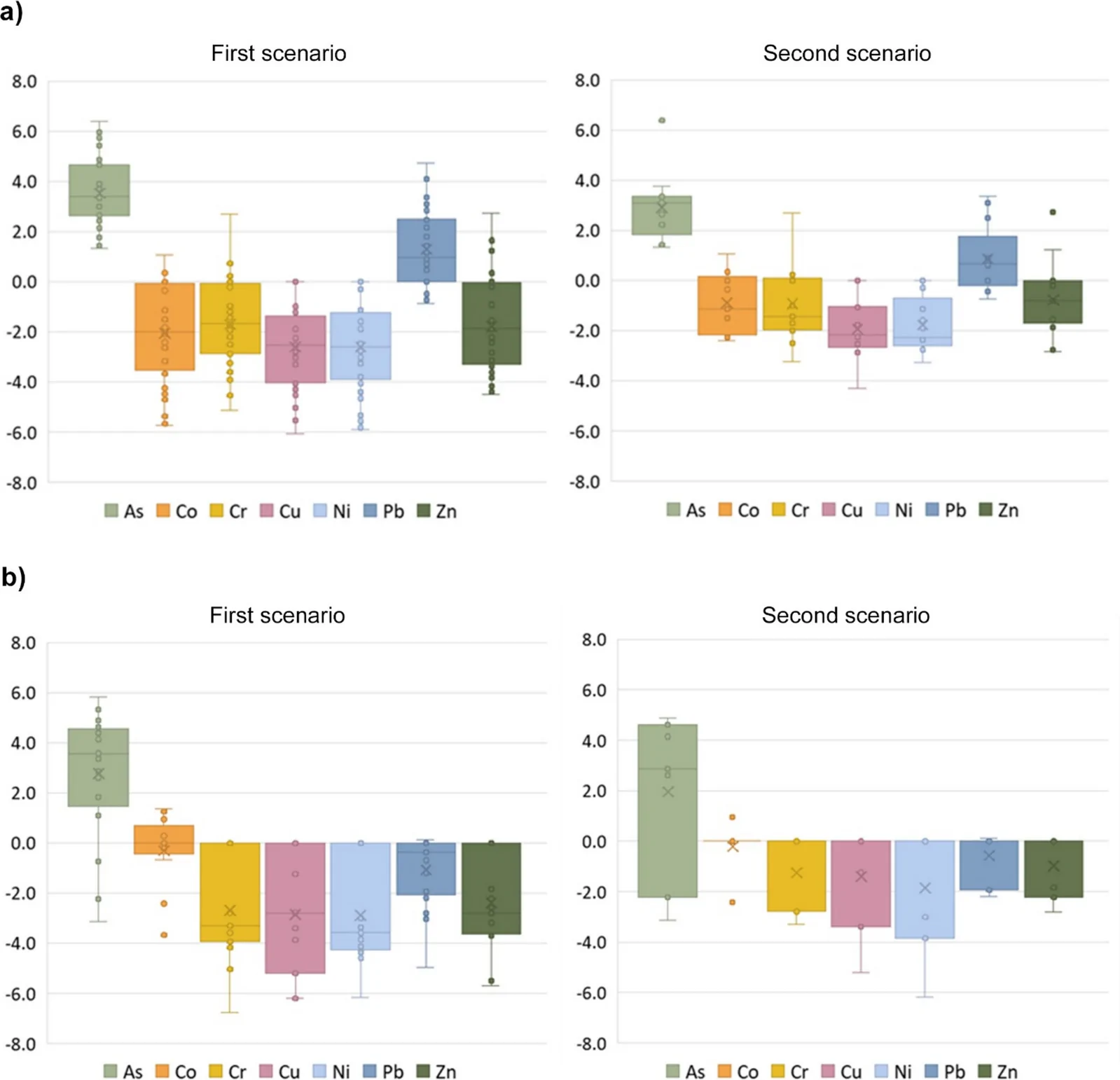
Box plots of Igeo values of soil samples at the Pestarena (**a**) and Crocette (**b**) site under the first and second classification scenarios

Regarding the soil samples from the Pestarena area, the Igeo values for the first scenario indicate a mean As value of 3.5, with some samples showing values exceeding 5.0 (Fig. 6a). This classifies the soils as heavily to extremely contaminated. Samples show moderate Pb contamination, with mean values of 1.5. Although some values for Co, Cr, Cu, Ni and Zn exceed zero, the soil are generally uncontaminated by these elements. In the second scenario, As and Pb remain the main contaminants with slightly lower mean values.

For the Crocette area the Igeo is similar for both scenarios (Fig. 6b). Arsenic is the main contaminant, with mean values falling within the moderately to heavily contaminated category (2.8 for the first scenario and 2.0 for the second scenario). Slight Co contamination was observed in some samples, however mean values remained within the non-contaminated range, as was also the case for Cr, Cu, Ni, Pb and Zn.

**Fig. 7:**
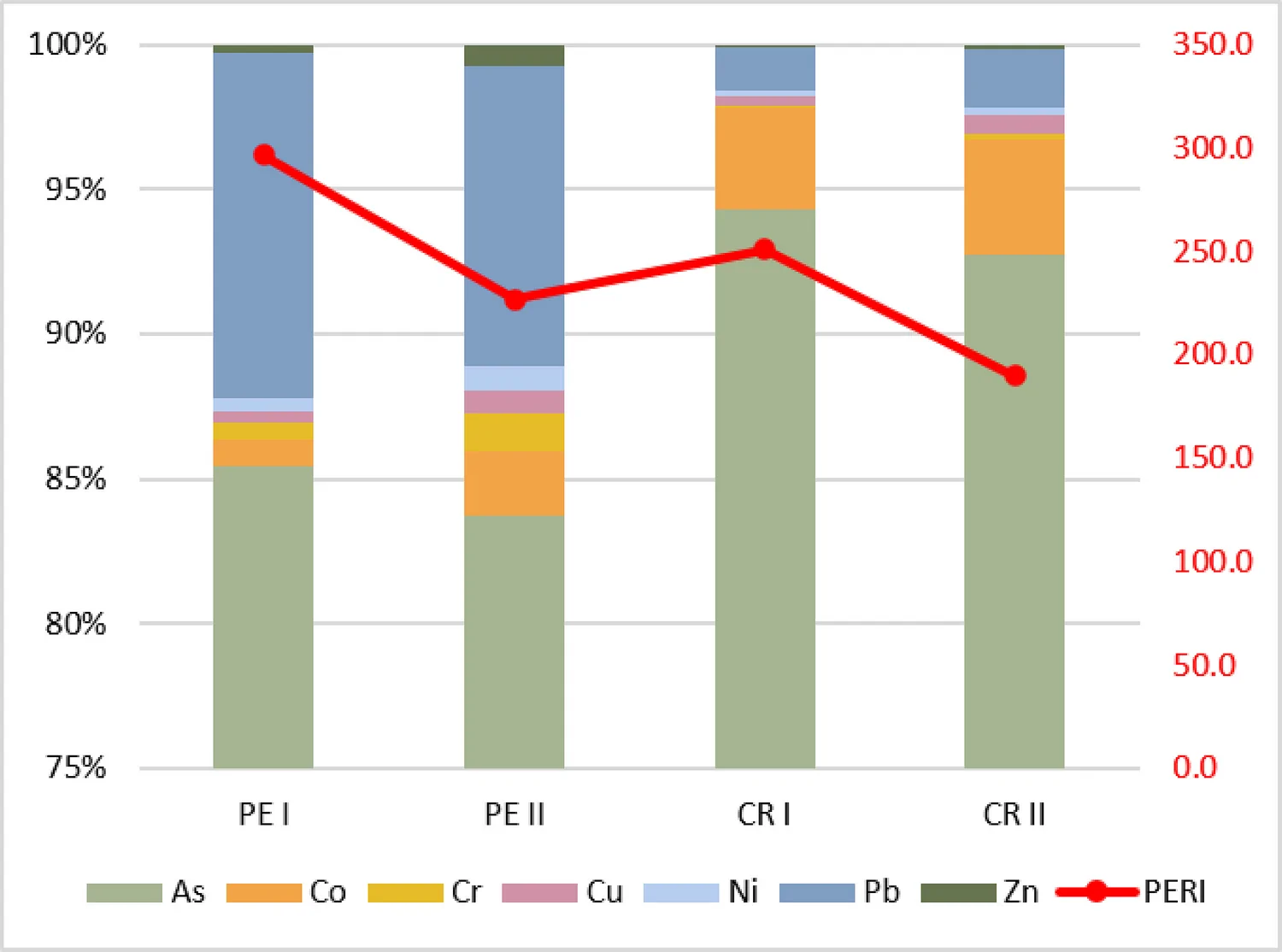
Potential Ecological Risk Index for Pestarena (PE) and Crocette (CR) soil samples under first (I) and second (II) scenarios. The bars show the percentage distribution of mean ERI values relative to the total PERI. PERI mean value is indicated by the red

Potential Ecological Risk Index for Pestarena (PE) and Crocette (CR) under the first (I) and second (II) scenarios was also calculated, where PERI represents the cumulative ecological risk obtained as the sum of the ERI for each PTE (Fig. 7).

At the Pestarena site, As is the dominant contributor to ERI, showing very high ecological risk in both scenarios. Pb contributes a significant to high risk in the first scenario and a high risk in the second. In both scenarios Co displays moderate risk in a limited number of samples, while Cr, Cu, Ni, Zn show low risk.

As a result of these contributions, the overall PERI indicates a moderate ecological risk under the first scenario, with a mean value of 296.4. Under the second scenario, mean PERI values are slightly lower but remain within the moderate risk category reflecting a slight reduction in As and Pb ERI values.

Similarly, at the Crocette site, As is the main driver of ERI. In the second scenario, the mean values for As are lower than in the first but still fall within the very high risk category. Co contributes moderate to significant risk, whereas Pb shows moderate risk only in a few samples. Consequently, PERI values indicate moderate ecological risk in both scenarios, with mean values of 251.1 and 189.4, respectively.

Summarising the results, the comparison of the two scenarios shows that Igeo values are generally equal or lower in the second, indicating a general reduction in contamination. Although both sites remain within the moderate ecological risk category, PERI values also decrease in the second scenario. Expanding the area classified as EW slightly reduces environmental impact but increases the volume of material that needs to be treated. Conventional remediation technologies offer limited advantages, focusing mainly on containment and stabilisation rather than valorisation. In contrast, treating EW as a resource enables RM and CRM recovery, while also reducing ecological risk and contamination.

### Potential recovery

To make a preliminary assessment of the potential for CRM recovery, the concentrations detected in the analysed samples were compared with values reported in studies on secondary resources such as EW. As they depend on site-specific factors, including the type of material, available recovery technologies, market conditions and economic parameters (Cairns and Shinkuma 2003), these reference values are not absolute thresholds, nevertheless they provide a useful benchmark. The focus was on As, Be, Bi, Sb, W and REE, which showed enrichment (based on the normalised concentration to the UCC). Key statistics compared with minimum reported values are summarised in Table 1, while a summary of the relevant literature data are provided in the Supplementary Information (Table S4).

**Table 1:** Concentrations of As, Be, Bi, Sb, W, and REE (mg/kg) in EW samples collected from the Crocette and Pestarena sites. For each element and site, the mean (MEAN), minimum (MIN), maximum (MAX) and standard deviation (STD) are reported. The 'Literature Reference' column shows the minimum concentrations reported in the literature for EW and is used as an initial threshold for recovery processes

		Unit	As	Be	Bi	Sb	W	∑REE
Pestarena	MEAN	mg/kg	15 386.8	2.7	20.2	8.6	76.2	185.8
	MIN	mg/kg	19.8	1.5	0.6	0.1	5.1	114.2
	MAX	mg/kg	78 726.3	3.1	55.3	36.6	146.0	231.6
Crocette	MEAN	mg/kg	20 505.7	6.1	3.1	8.6	83.6	109.6
	MIN	mg/kg	426.0	4.1	0.3	0.5	15.9	41.6
	MAX	mg/kg	89 110.9	13.1	7.2	19.9	159.0	134.3
Literature reference	MIN	mg/kg	330.0	140.0	180.0	800.0	480.0	580.0
			(Choi et al. 2016)	(Lohmeier et al. 2021)	(Radu et al. 2024)	(Lemos et al. 2023b)	(Han et al. 2025)	(Fleming et al. 2021)

Arsenic is present at high levels, suggesting substantial recovery potential, whereas Be, Bi, Sb, W, and REE generally occur at concentrations below the thresholds reported in these studies. This does not preclude the possibility of improving extraction efficiency or exploring the simultaneous recovery of multiple elements, which could enhance overall economic viability. Nevertheless, the presence of these elements in EW is noteworthy and warrants further investigation to develop valorisation strategies for their potential recovery as associated CRMs.

Examples of the concurrent recovery of As, Sb, Bi, and W from different types of mining and processing residues have been reported in the literature. Case studies are briefly summarised below, with further details provided in the Supplementary Information. Recovery of As and Sb has been reported from Sb flotation waste via acid leaching (Dembele et al. 2024), and from Cu smelter flue dusts via acid chloride leaching, with subsequent hydrolysis to precipitate Sb and Bi (Zhang et al. 2012) or As reduction and Sb recovery (Xue et al. 2021). Copper electrorefining residues are treated to stabilise As as scorodite and selective precipitate of Sb and Bi as oxychlorides (Luo et al. 2023a). Similarly, copper smelter dust is processed through hydrometallurgical leaching and selective precipitation to achieve Bi recovery as a oxychloride precipitate, while concurrently generating stabilized As (Córdoba et al. 2026). From W-bearing mineral residues, different hydrometallurgical processes have been applied to recover W and As: alkaline leaching with pressure oxidation and organic solvent extraction (Wu et al. 2023), deep eutectic solvent leaching combined with electrodialysis (Almeida et al. 2020), and acid leaching followed by extraction, precipitation, reduction to recover W as an ultrafine powder and As in elemental form (Cao et al. 2024).

A range of processes to recover As in association with Sb, Bi and W are illustrated by these studies. However, the recovery efficiency is strongly dependent on the characteristics of the initial residue, which emphasises the need for more detailed characterisation of EW and further evaluation of the most sustainable technologies to be applied. Furthermore, As represents a particular challenge for its recovery because it simultaneously constitutes a valuable secondary resource and a major environmental and health risk. As a CRM, As is relevant for several specialised applications, particularly in gallium arsenide (GaAs) semiconductors and other electronic and photovoltaic technologies (USGS 2021; European Commission: Directorate-General for Internal Market et al. 2023b; SFA 2026). At the same time, the high toxicity and mobility of As make its long-term management a major environmental concern, as exposure may occur through contaminated water, food and industrial processes and can result in severe chronic health effects (ATSDR 2007; WHO 2022). The recovery of commercially viable As products and co-associated metals should therefore be coupled with the safe management of the As remaining after processing. This residual As fraction may require appropriate stabilisation strategies, such as precipitation as scorodite or vitrification, to prevent its long-term release into the environment (Lemos et al. 2023a; Luo et al. 2023b; Sun et al. 2026). The environmental sustainability of the complete system must therefore consider both the recovery of As from the EW and the long-term behaviour and stability of the As residues, as well as the environmental and economic liability associated with the storage and management of As waste (Zhang et al. 2021; Córdoba et al. 2026). Future studies should therefore further investigate the economic aspects associated with the production process and the related safety requirements, which were beyond the scope of the present research, in order to provide a more comprehensive assessment of the overall economic sustainability of the proposed approach.

#### Estimation of recoverable CRMs

To complete the characterisation, it is also important to estimate the volumes of EW to be treated and the quantity of residual CRMs. This improves the understanding of the value of the resources and enables to conduct an initial economic assessment, assuming the recovery of As, Bi, Sb, and W. To perform the volume calculations, it was necessary to identify and define the areas of the dumps. At Pestarena, the first scenario includes one WR dump, three TA dumps and two TA concrete basins. In the second scenario, areas adjacent to the TA dumps and concrete basins are added. At Crocette, the first scenario includes one TA concrete basin and one TA dump. The second scenario adds one inferred TA dump and one inferred WR dump. Deposit thicknesses were estimated based on visual observations and available stratigraphic data from ARPA Piemonte. The quantity (kg) of residual CRMs (see Eq. 5 and Eq. 6) and their theoretical economic value (€) (see Eq. 7 and Eq. 8) were also calculated and reported in Table 2.

**Table 2:** Estimated volumes (m^3^) of EW, quantities of residual CRMs (kg), and economic values (€) estimated assuming 100%, 80%, 70%, and 60% processing recovery at Pestarena and Crocette under the first (I) and second (II) scenarios

Pestarena	Scenario I	EW volume (m^3^)		CRM quantity (kg)	Theoretical total EV(€)	Total EV (€)	Total EV (€)	Total EV (€)
					100%	80%	70%	60%
		7 413.6	As	239176.0	38 3317.5	30 6654.0	268 322.3	229 990.5
			Bi	36.2				
			Sb	100.3				
			W	975.1				
	Scenario II	18 341.0	As	642546.1	1 029 779.3	823 823.4	72 0845.5	617 867.6
			Bi	97.1				
			Sb	269.5				
			W	2619.6				
Crocette	Scenario I	968.0	As	26810.0	43 901.0	35120.8	30730.7	26 340.6
			Bi	35.2				
			Sb	15.0				
			W	132.8				
	Scenario II	5878.0	As	160675.1	263 104.6	210 483.7	184 173.2	157 862.8
			Bi	210.9				
			Sb	89.8				
			W	795.7				

These values are preliminary estimates based on the assumption of 60%, 70%, 80% and 100% recovery. Reported recovery and extraction efficiencies for comparable secondary resources range from 77–99% for W (Almeida et al. 2020; Wu et al. 2023; Cao et al. 2024), 81–99% for As (Almeida et al. 2020; Xue et al. 2021; Wu et al. 2023; Dembele et al. 2024), up to 98% for Sb (Dembele et al. 2024), and 76–99% for Bi (Khmelnitskaya and Beskrovnaya 2007; Córdoba et al. 2026) (see Supplementary Information). Considering the variability associated with the characteristics of the raw material, mineralogical associations, pre-treatment and processing technologies, a conservative recovery range of 60–80% was therefore adopted for the evaluation of the theoretical economic values, with 100% considered as a theoretical upper bound rather than an expected metallurgical recovery. As expected, higher CRM quantities and values are obtained under the second scenario, which considers larger volumes. While As is the most abundant CRM, Bi, Sb and W, are also present in significant quantities. This estimation provides an initial understanding of the scale of available resources and the potential for future recovery using efficient technologies; however, is not sufficient to assess the economic feasibility of potential exploitation. Several cost components must be considered: i) remediation of the areas (partially offset by CRM recovery revenues); ii) processing of EW; iii) disposal of new residues generated during reprocessing. Only by considering both the costs of conventional remediation and the costs associated with EW processing for CRM recovery can a realistic economic evaluation of this approach be made. In addition, environmental and social impacts may also occur during processing and should be assessed.

## Conclusion

This study investigates the environmental impact and potential as secondary resources of EW from former Au mines in the Anzasca Valley (NW Italy). The study carries out a comprehensive characterisation of EW with the aim of providing an integrated understanding of their current state of EW produced from historical mining operations. To address uncertainties in paste EW management, two scenarios were applied, reflecting different EW volumes and dispersion.

Geochemical analyses showed enrichment of CRMs. Both mining sites denoted very high As enrichment, followed by W, Sb, and Bi, and a slight enrichment of Be and some REE at Pestarena (La, Ce, Pr, Nd). Environmental impacts assessment showed that As is the main pollutant at both Pestarena and Crocette, with Pestarena also showing moderate Pb contamination. PERI indicated an overall moderate ecological risk, driven mainly by As, with contributions from Pb and Co. In the first scenario, the Igeo and PERI values were both higher. Conversely, expanding the EW area in the second scenario slightly reduced the Igeo and PERI values, but increased the volume of material requiring management. This suggests that treating EW as a secondary resource could provide a sustainable waste management within remediation projects.

Assessment of the potentially recoverable CRMs from EW indicates that As occurs in elevated concentrations, making it the main element with recovery potential while Be, Bi, Sb, W and REEs are generally present at levels below the economic thresholds defined for primary or secondary deposits, although their presence is significant. Several treatment processes in literature demonstrated the feasibility which enable the concurrent recovery of As, Sb, Bi and W from mining residues. Preliminary estimates of EW volumes enable an approximate quantification of the residual contents of As, Bi, Sb, and W, thereby allowing an evaluation of their economic value and indicating that significant quantities of CRMs are still present for potential recovery.

The results suggest that, rather than being managed as waste to be removed or stabilised, abandoned EW can be reframed as secondary deposits. They can provide the strategic materials that are essential for the EU’s green and digital transition. This integrated approach provides a more sustainable alternative to conventional interventions for contaminated waste by aligning he mitigation of environmental impacts with resource recovery and supply security. Further research is required to characterise these materials more accurately, study efficient treatment processes and evaluate the economic and environmental sustainability of potential recovery strategies.

## Supplementary Information

Below is the link to the electronic supplementary material.

ESM 1 (DOCX 2.57 MB)

## Data Availability

Data will be available upon request

## Abbreviations

AMD: Acid mine drainage
CEAP: Circular Economy Action Plan
CF: Contamination factor
CR: Crocette
CRM: Critical Raw Material
ERI: Ecological risk index
EW: Extractive waste
EWF: Extractive waste facility
Igeo: Geo-accumulation index
PE: Pestarena
PERI: Potential ecological risk index
PTE: Potentially toxic element
REE: Rare Earth Element
RM: Raw material
TA: Tailings
UCC: Upper continental crust
WR: Waste rock

## Declarations

### Clinical trial number

This is not applicable.

## References

[CR1] Actlabs (2026) 4-Acid “Near Total” Digestion. In: Activation Laboratories Ltd. (Actlabs). https://actlabs.com/geochemistry-analysis/exploration-geochemistry/4-acid-near-total-digestion/. Accessed 20 Aug 2026

[CR2] Adiansyah JS, Rosano M, Vink S, Keir G (2015) A framework for a sustainable approach to mine tailings management: disposal strategies. J Clean Prod 108:1050–1062. 10.1016/j.jclepro.2015.07.139

[CR3] Allegretta I, Porfido C, Martin M, Barberis E, Terzano R, Spagnuolo M (2018) Characterization of As-polluted soils by laboratory X-ray-based techniques coupled with sequential extractions and electron microscopy: the case of Crocette gold mine in the Monte Rosa mining district (Italy). Environ Sci Pollut Res 25:25080–25090. 10.1007/s11356-018-2526-9 29936615

[CR4] Almeida J, Craveiro R, Faria P, Silva AS, Mateus EP, Barreiros S, Paiva A, Ribeiro AB (2020) Electrodialytic removal of tungsten and arsenic from secondary mine resources — deep eutectic solvents enhancement. Sci Total Environ 710:136364. 10.1016/j.scitotenv.2019.136364 31926419

[CR5] Amos RT, Blowes DW, Bailey BL, Sego DC, Smith L, Ritchie AIM (2015) Waste-rock hydrogeology and geochemistry. Appl Geochem 57:140–156. 10.1016/j.apgeochem.2014.06.020

[CR6] ARPA Piemonte (2019) Diffuse soil contamination. In: State of the Environment in Piedmont - 2019 Report. https://relazione.ambiente.piemonte.it/2019/it/territorio/stato/suolo-contaminazione. Accessed 21 Jul 2025 (in italian)

[CR7] Assawincharoenkij T, Hauzenberger C, Ettinger K, Sutthirat C (2018) Mineralogical and geochemical characterization of waste rocks from a gold mine in northeastern Thailand: application for environmental impact protection. Environ Sci Pollut Res 25:3488–3500. 10.1007/s11356-017-0731-6 29159434

[CR8] ATSDR (2007) Toxicological profile for arsenic. U.S. Department of Health and Human Services, Public Health Service, Agency for Toxic Substances and Disease Registry, Atlanta. https://www.atsdr.cdc.gov/toxprofiles/tp2.pdf. Accessed 15 Jul 2025

[CR9] Aznar-Sánchez JA, García-Gómez JJ, Velasco-Muñoz JF, Carretero-Gómez A (2018) Mining waste and its sustainable management: advances in worldwide research. Minerals (Basel) 8:284. 10.3390/min8070284

[CR10] Barcelos DA, Pontes FVM, da Silva FANG, Castro DC, dos Anjos NOA, Castilhos ZC (2020) Gold mining tailing: environmental availability of metals and human health risk assessment. J Hazard Mater 397:122721. 10.1016/j.jhazmat.2020.122721 32473498

[CR11] Bauer M, Blodau C (2006) Mobilization of arsenic by dissolved organic matter from iron oxides, soils and sediments. Sci Total Environ 354:179–190. 10.1016/j.scitotenv.2005.01.027 16398994

[CR12] Bidone E, Castilhos Z, Cesar R, Santos MC, Sierpe R, Ferreira M (2016) Hydrogeochemistry of arsenic pollution in watersheds influenced by gold mining activities in Paracatu (Minas Gerais State, Brazil). Environ Sci Pollut Res Int 23:8546–8555. 10.1007/s11356-016-6089-3 26797944

[CR13] BRGM (2001) Management of mining, quarrying and ore-processing waste in the European Union. Report BRGM/RP-50319-FR, Bureau de Recherches Géologiques et Minières (BRGM), Orléans. https://ec.europa.eu/environment/pdf/waste/studies/mining/0204finalreportbrgm.pdf. Accessed 23 Jul 2025

[CR14] Bruck R (1985) The Pestarena gold mine and the other Italian gold deposits. Comunità montana Valle Anzasca, Domodossola

[CR15] Bruno C (2005) Geochemical and mineralogical behaviour of arsenic in the area surrounding the gold mines of Anzasca Valley. Master thesis, University of Turin, Turin (in italian)

[CR16] Cairns RD, Shinkuma T (2003) The choice of the cutoff grade in mining. Resour Policy 29:75–81. 10.1016/j.resourpol.2004.06.002

[CR17] Candeias C, Ávila P, Coelho P, Teixeira JP (2019) Mining activities: health impacts. In: Nriagu J (ed) Encyclopedia of environmental health (second edition). Elsevier, Oxford, pp 415–435. 10.1016/B978-0-12-409548-9.11056-5

[CR18] Cao C, Fu A, Mai Q, Li Y, Nie H, Wang J, Gong D, Qiu X (2024) Study on the integrated technology of arsenic removal and tungsten extraction based on the formation and decomposition of arsenotungstic acid. Chem Eng J 499:156963. 10.1016/j.cej.2024.156963

[CR19] Carvalho PCS, Neiva AMR, Silva MMVG, da Silva EAF (2014) Geochemical comparison of waters and stream sediments close to abandoned Sb-Au and As-Au mining areas, northern Portugal. Geochemistry (English Transl) 74:267–283. 10.1016/j.chemer.2013.08.003

[CR20] Caviglia C, Destefanis E, Masciocco L, Re D (2015) Environmental problems related to the presence of arsenic in the Anza Valley (Piedmont, North-Western Italy). In: Lollino G, Arattano M, Rinaldi M, Giustolisi O, Marechal JC, Grant G (eds) Engineering geology for society and territory - volume 3. Springer International Publishing, Cham, pp 421–424. 10.1007/978-3-319-09054-2_87

[CR21] Cerri R, Fantoni R (2017) The gold of Monte Rosa. Mining activity in the Alps: the future of a thousand-year history. CAI Sezione di Varallo, Gorno (BG), pp 9–24

[CR22] Chipera SJ, Bish DL (2002) FULLPAT : a full-pattern quantitative analysis program for X-ray powder diffraction using measured and calculated patterns. J Appl Crystallogr 35:744–749. 10.1107/S0021889802017405

[CR23] Choi J, Lee E, Choi SQ, Lee S, Han Y, Kim H (2016) Arsenic removal from contaminated soils for recycling via oil agglomerate flotation. Chem Eng J 285:207–217. 10.1016/j.cej.2015.09.105

[CR24] Clark AJ, Labaj AL, Smol JP, Campbell LM, Kurek J (2021) Arsenic and mercury contamination and complex aquatic bioindicator responses to historical gold mining and modern watershed stressors in urban Nova Scotia, Canada. Sci Total Environ. 10.1016/j.scitotenv.2021.147374 34045077

[CR25] Coelho P, Silva S, Roma-Torres J, Costa C, Henriques A, Teixeira J, Gomes M, Mayan O (2007) Health impact of living near an abandoned mine - case study: Jales mines. Int J Hyg Environ Health 210:399–402. 10.1016/j.ijheh.2007.01.004 17321206

[CR26] Córdoba P, Mayor L, Espí JJ, Licon E (2026) Addressing sustainability trade-offs in critical raw material recovery from metallurgical residues: An integrated environmental–economic–strategic framework. Clean Eng Technol 32:101226. 10.1016/j.clet.2026.101226

[CR27] Craw D, Cavanagh J, Druzbicka J, Harding JS, Kerr G, Pope J, Trumm D (2015) A Geoenvironmental Model for Orogenic Gold Deposits to Predict Potential Environmental Effects. Mine Water Environ 34:388–403. 10.1007/s10230-015-0358-0

[CR28] Craw D, Chappell DA (2000) Metal redistribution in historic mine wastes, Coromandel Peninsula, New Zealand. N Z J Geol Geophys 43:187–198. 10.1080/00288306.2000.9514880

[CR29] Craw D, Pope J (2017) Time-series monitoring of water–rock interactions in mine wastes, Macraes gold mine, New Zealand. N Z J Geol Geophys 60:159–175. 10.1080/00288306.2017.1307231

[CR30] Curti E (1987) Lead and oxygen isotope evidence for the origin of the Monte Rosa gold lode deposits (Western Alps, Italy); a comparison with Archean lode deposits. Econ Geol 82:2115–2140. 10.2113/gsecongeo.82.8.2115

[CR31] Dai S, Graham IT, Ward CR (2016) A review of anomalous rare earth elements and yttrium in coal. Int J Coal Geol 159:82–95. 10.1016/j.coal.2016.04.005

[CR32] Dantas SVA, Felix CSA, Cunha FAS, de Andrade JB, Ferreira SLC (2025) Risk assessment of mercury in soil, water, and sediments collected in a region impacted by gold mining. ACS Omega 10:8152–8159. 10.1021/acsomega.4c09617 PMC11886748 40060868

[CR33] Dedieu N, Allard L, Vigouroux R, Brosse S, Céréghino R (2014) Physical habitat and water chemistry changes induced by logging and gold mining in French Guiana streams. Knowl Manag Aquat Ecosyst. 10.1051/kmae/2014026

[CR34] Dembele S, Akcil A, Panda S (2024) Investigation of the characteristics of stibnite (Sb_2_S_3_) flotation tailings and extraction of critical metals (Sb and As): optimization and scale-up. Miner Eng 216:108883. 10.1016/j.mineng.2024.108883

[CR35] Di Duca F, Montuori P, De Rosa E, De Simone B, Russo I, Nubi R, Triassi M (2024) Assessing heavy metals in the Sele River Estuary: an overview of pollution indices in Southern Italy. Toxics 12:38. 10.3390/toxics12010038 PMC10819315 38250994

[CR36] Dino GA, Mehta N, Rossetti P, Ajmone-Marsan F, De Luca DA (2018) Sustainable approach towards extractive waste management: two case studies from Italy. Resour Policy 59:33–43. 10.1016/j.resourpol.2018.07.009

[CR37] European Commission: Directorate-General for Communication (2020) Circular economy action plan – the European Green Deal. Publications Office. 10.2775/458852

[CR38] European Commission: Directorate-General for Internal Market, Industry, Entrepreneurship and SMEs (2023) European critical raw materials act. Publications Office of the European Union. 10.2873/076637

[CR39] European Commission: Directorate-General for Internal Market, Industry, Entrepreneurship and SMEs (2023) Study on the critical raw materials for the EU 2023 – final report. Publications Office of the European Union. 10.2873/725585

[CR40] Eurostat (2024) Waste statistics. In: Eurostat Statistics Explained. https://ec.europa.eu/eurostat/statistics-explained/index.php?title=Waste_statistics. Accessed 25 Jul 2025

[CR41] da Silva E. Ferreira, Zhang C, Serrano Pinto L, Patinha C, Reis P (2004) Hazard assessment on arsenic and lead in soils of Castromil gold mining area, Portugal. Appl Geochem 19:887–898. 10.1016/j.apgeochem.2003.10.010

[CR42] Fleming P, Orrego P, Pinilla F (2021) Recovery of rare earth elements present in mining tails, by leaching with nitric and hydrochloric solutions. World J Nucl Sci Technol 11:1–16. 10.4236/wjnst.2021.111001

[CR43] Force ER (1991) Geology of titanium-mineral deposits. Geol Soc Am Bull. 10.1130/spe259-p1

[CR44] Garbarino E, Orveillon G, Saveyn HGM (2020) Management of waste from extractive industries: The new European reference document on the Best Available Techniques. Resour Policy 69:101782. 10.1016/j.resourpol.2020.101782

[CR45] Gomes P, Valente T (2024) Seasonal impact of acid mine drainage on water quality and potential ecological risk in an old sulfide exploitation. Environ Sci Pollut Res 31:21124–21135. 10.1007/s11356-024-32367-1 PMC10948584 38388972

[CR46] Guerrieri N, Lami A, Musazzi S, Austoni M, Marchetto A, Orrù A, Giacomotti P, Tartari G, Lucchini G, Scaglioni L, Borgonovo G (2023) Arsenic distribution and pollution in three mountain streams (Anzasca Valley, Italian Central Alps). Sustainability (Basel) 15:14217. 10.3390/su151914217

[CR47] Hakanson L (1980) An ecological risk index for aquatic pollution control. A sedimentological approach. Water Res 14:975–1001. 10.1016/0043-1354(80)90143-8

[CR48] Han Z, Edraki M, Golev A (2025) Characterization, potential valuable metals recovery and remediation of historic wolframite mining waste. Sep Purif Technol 354:129009. 10.1016/j.seppur.2024.129009

[CR49] Hou Y, Zhao Y, Lu J, Wei Q, Zang L, Zhao X (2023) Environmental contamination and health risk assessment of potentially toxic trace metal elements in soils near gold mines – a global meta-analysis. Environ Pollut 330:121803. 10.1016/j.envpol.2023.121803 37187277

[CR50] Jamieson HE, Walker SR, Parsons MB (2015) Mineralogical characterization of mine waste. Appl Geochem 57:85–105. 10.1016/j.apgeochem.2014.12.014

[CR51] Jordens A, Cheng YP, Waters KE (2013) A review of the beneficiation of rare earth element bearing minerals. Miner Eng 41:97–114. 10.1016/j.mineng.2012.10.017

[CR52] Kalisz S, Kibort K, Mioduska J, Lieder M, Małachowska A (2022) Waste management in the mining industry of metals ores, coal, oil and natural gas - a review. J Environ Manage 304:114239. 10.1016/j.jenvman.2021.114239 34902687

[CR53] Khmelnitskaya OD, Beskrovnaya VP (2007) A technology of precious metals recovery from gold-bismuth ores. In: Proceedings of World Gold 2007, Cairns, Australia, pp 245–250.

[CR54] Laker MC (2023) Environmental impacts of gold mining—with special reference to South Africa. Mining 3:205–220. 10.3390/mining3020012

[CR55] Larkins C, Turunen K, Mänttäri I, Lahaye Y, Hendriksson N, Forsman P, Backnäs S (2018) Characterization of selected conservative and non-conservative isotopes in mine effluent and impacted surface waters: Implications for tracer applications at the mine-site scale. Appl Geochem 91:1–13. 10.1016/j.apgeochem.2018.01.005

[CR56] Lattanzi P, Curti E, Bastogi M (1989) Fluid inclusion studies on the gold deposits of the upper Anzasca Valley, northwestern Alps, Italy. Econ Geol 84:1382–1397. 10.2113/gsecongeo.84.5.1382

[CR57] Lemos MG, Valente TM, Marinho Reis AP, Ferreira Fonseca RM, Guabiroba F, da Mata Filho JG, Magalhães MF, Delbem ID, Rebelo Diório G (2023) Adding value to mine waste through recovery Au, Sb, and As: the case of auriferous tailings in the Iron Quadrangle, Brazil. Minerals. 10.3390/min13070863

[CR58] Li Y, Yang J, Zhao Z (2017) Recovery of tungsten and molybdenum from low-grade scheelite. JOM 69:1958–1962. 10.1007/s11837-017-2440-5

[CR59] Lindsay MBJ, Moncur MC, Bain JG, Jambor JL, Ptacek CJ, Blowes DW (2015) Geochemical and mineralogical aspects of sulfide mine tailings. Appl Geochem 57:157–177. 10.1016/j.apgeochem.2015.01.009

[CR60] Lohmeier S, Lottermoser BG, Schirmer T, Fuchsloch W (2021) Reprocessing potential of pegmatite tailings for rare metal extraction and brick fabrication, Uis, Namibia. South Afr J Geol 124:639–662. 10.25131/sajg.124.0015

[CR61] Lottermoser B (2010) Mine Wastes. Springer, Berlin, Heidelberg. 10.1007/978-3-642-12419-8

[CR62] Luo D, Fernández de Labastida M, Cortina JL, Lopez J (2023) Recovery of antimony and bismuth from arsenic-containing waste streams from the copper electrorefining circuit: An example of promoting critical metals circularity from secondary resources. J Clean Prod 415:137902. 10.1016/j.jclepro.2023.137902

[CR63] Machairas E, Varouchakis EA, Sfakianakis M, Rozakis S, Deveci M, Galetakis M (2025) Cost – benefit analysis through stochastic risk assessment on mining waste management considering the circular economy’s requirements. J Clean Prod 489:144388. 10.1016/j.jclepro.2024.144388

[CR64] Mancini S, Casale M, Tazzini A, Dino GA (2024) Use and recovery of extractive waste and tailings for sustainable raw materials supply. Mining 4:149–167. 10.3390/mining4010010

[CR65] Mourinha C, Palma P, Alexandre C, Cruz N, Rodrigues SM, Alvarenga P (2022) Potentially toxic elements’ contamination of soils affected by mining activities in the Portuguese sector of the Iberian Pyrite Belt and optional remediation actions: a review. Environments (Basel) 9:11. 10.3390/environments9010011

[CR66] Muller G (1969) Index of geoaccumulation in sediments of the Rhine river. GeoJournal 2:108–118

[CR67] Munford KE, Gilbert-Parkes S, Mykytczuk NCS, Basiliko N, Yakimovich KM, Poulain A, Watmough SA (2023) How arsenic contamination influences downslope wetland plant and microbial community structure and function. Sci Total Environ. 10.1016/j.scitotenv.2023.162839 36921856

[CR68] Nepote Valentin G (2007) Arsenic contamination in an area surrounding a gold mine (Pestarena, Anzasca Valley). Master thesis, University of Turin, Turin (in italian)

[CR69] Ngole-Jeme VM, Fantke P (2017) Ecological and human health risks associated with abandoned gold mine tailings contaminated soil. PLoS One 12:e0172517. 10.1371/journal.pone.0172517 PMC5319768 28222184

[CR70] Passariello B, Giuliano V, Quaresima S, Barbaro M, Caroli S, Forte G, Carelli G, Iavicoli I (2002) Evaluation of the environmental contamination at an abandoned mining site. Microchem J 73:245–250. 10.1016/S0026-265X(02)00069-3

[CR71] Piana F, Fioraso G, Irace A, Mosca P, d’Atri A, Barale L, Falletti P, Monegato G, Morelli M, Tallone S, Vigna GB (2017) Geology of Piemonte region (NW Italy, Alps–Apennines interference zone). J Maps 13:395–405. 10.1080/17445647.2017.1316218

[CR72] Radu V-M, Dinca G, Ivanov A-A, Szabo R, Cetean VM (2024) New data regarding the identification of critical raw materials recoverable from raw, processed and the waste mining industry materials from Romania. Environ Sci Pollut Res 31:40592–40608. 10.1007/s11356-023-26536-x 36991209

[CR73] Ritcey GM (2005) Tailings management in gold plants. Hydrometallurgy 78:3–20. 10.1016/j.hydromet.2005.01.001

[CR74] Rolando A (2008) Inorganic pollutants around the Pestarena gold mine (Anzasca Valley) and proposal for remediation intervention. Master thesis, University of Turin, Turin (in italian)

[CR75] Rudnick RL, Gao S (2014) Composition of the continental crust. In: Holland HD, Turekian KK (eds) Treatise on geochemistry (second edition). Elsevier, Oxford, pp 1–51. 10.1016/B978-0-08-095975-7.00301-6

[CR76] Sanchez AA, Jackisch C, Oelschlägel M, Hedrich S, Lau MP (2025) Harmful metal export from abandoned mines controlled by hydrodynamic and biogeochemical drivers. ACS EST Water 5:5184–5194. 10.1021/acsestwater.5c00334

[CR77] Santos AS, Braz BF, Sanjad P, Cruz ACR, Crapez MAC, Neumann R, Santelli RE, Keim CN (2023) Role of indigenous microorganisms and organics in the release of iron and trace elements from sediments impacted by iron mine tailings from failed Fundão dam. Environ Res 220:115143. 10.1016/j.envres.2022.115143 36574804

[CR78] SFA (2026) The arsenic market. In: SFA Oxford. https://www.sfa-oxford.com/rare-earths-and-minor-metals/minor-metals-and-minerals/arsenic-33-as/. Accessed 20 Aug 2026

[CR79] Singh S, Ram LC, Masto RE, Verma SK (2011) A comparative evaluation of minerals and trace elements in the ashes from lignite, coal refuse, and biomass fired power plants. Int J Coal Geol 87:112–120. 10.1016/j.coal.2011.05.006

[CR80] Spitz K, Trudinger J (2019) Mining and the environment: from ore to metal, 2nd edn. CRC Press, London. 10.1201/9781351183666

[CR81] Sracek O, Choquette M, Gélinas P, Lefebvre R, Nicholson RV (2004) Geochemical characterization of acid mine drainage from a waste rock pile, Mine Doyon, Québec, Canada. J Contam Hydrol 69:45–71. 10.1016/S0169-7722(03)00150-5 14972437

[CR82] Sun Y, Yang T, Zhang H, Peng Q, Xiong W, Tang H, Liu Y, Zou Z, Lei K (2026) Research progress in the synthesis and process application of scorodite. Pol J Environ Stud 35:15–27. 10.15244/pjoes/195782

[CR83] Tayebi-Khorami M, Edraki M, Corder G, Golev A (2019) Re-thinking mining waste through an integrative approach led by circular economy aspirations. Minerals. 10.3390/min9050286

[CR84] US EPA (1996) Method 3050B: Acid Digestion of Sediments, Sludges, and Soils

[CR85] USGS 2021 Minerals Yearbook: Arsenic. U.S. Geological Survey, Reston, VA. https://pubs.usgs.gov/myb/vol1/2021/myb1-2021-arsenic.pdf. Accessed 23 Jul 2025

[CR86] Valente TM, Antunes MD, Braga MAS, Pamplona JM (2011) Geochemistry and mineralogy of ochre-precipitates formed as waste products of passive mine water treatment. Geochem Explor Environ Anal 11:103–106. 10.1144/1467-7873/09-IAGS-009

[CR87] Venkateswarlu K, Nirola R, Kuppusamy S, Thavamani P, Naidu R, Megharaj M (2016) Abandoned metalliferous mines: ecological impacts and potential approaches for reclamation. Rev Environ Sci Biotechnol 15:327–354. 10.1007/s11157-016-9398-6

[CR88] WHO (2022) Arsenic. World Health Organization. https://www.who.int/news-room/fact-sheets/detail/arsenic. Accessed 20 Aug 2026

[CR89] Wu X, Qing J, Li J, Zhang G, Wu S, Zeng L, Li Q, Cao Z, Wang M, Guan W (2023) A green process for arsenic removal and tungsten recovery from tungsten residue waste: focused on the separation and recovery of arsenic and tungsten via solvent extraction followed by H_2_S precipitation. J Environ Chem Eng 11:110952. 10.1016/j.jece.2023.110952

[CR90] Xue J, Long D, Zhong H, Wang S, Liu L (2021) Comprehensive recovery of arsenic and antimony from arsenic-rich copper smelter dust. J Hazard Mater 413:125365. 10.1016/j.jhazmat.2021.125365 33930948

[CR91] Yang C, Li S, Liu R, Sun P, Liu K (2015) Effect of reductive dissolution of iron (hydr)oxides on arsenic behavior in a water–sediment system: first release, then adsorption. Ecol Eng 83:176–183. 10.1016/j.ecoleng.2015.06.018

[CR92] Zaniboni L, Dino GA, Lasagna M, Antunes IMHR, De Luca DA (2025) post-mining water-soil interaction in au-mine area: Piedmont region (NW Italy) case studies. In: Valente T, Muhlbauer R, Ordonez A, Wolkersdorfer C (eds) Proceedings of the International Mine Water Association Conference, Imwa 2025. Int Mine Water Assoc, Wendelstein, pp 1035–1041

[CR93] Zhang B, Li Q, Shen W, Min X (2012) Recovery of bismuth and antimony metals from pressure-leaching slag. Rare Met 31:102–106. 10.1007/s12598-012-0471-1

[CR94] Zhang H, Yu J, Zhou S (2014) Spatial distribution of As, Cr, Pb, Cd, Cu, and Zn in the water and sediment of a river impacted by gold mining. Mine Water Environ 33:206–216. 10.1007/s10230-013-0254-4

[CR95] Zhang W, Che J, Xia L, Wen P, Chen J, Ma B, Wang C (2021) Efficient removal and recovery of arsenic from copper smelting flue dust by a roasting method: process optimization, phase transformation and mechanism investigation. J Hazard Mater 412:125232. 10.1016/j.jhazmat.2021.125232 33951866

