# Supplementary material for: From environmental burden to resource potential: environmental impact evaluation and Critical Raw Materials recovery from abandoned gold mining waste in the Western Alps (NW Italy)
Source: Environ Sci Pollut Res Int. 2026 Sep 26;33(31):16042–61. doi: 10.1007/s11356-026-38241-6 (PMC13642380; doi:10.1007/s11356-026-38241-6)
Supplement: Supplementary file 1 [file 11356_2026_38241_MOESM1_ESM.docx]

**Supplementary Information**

S1. **Samples classification**

Table S1 Sample location, ID, coordinates (ESPG: 32632), material type (waste rock, tailings, soil, sediments), source of the data (previous studies samples or new samples) and classification code: 1 for indicated EW, 2 for inferred EW (considered as contaminated mine soil in the conservative scenario and as EW in the extended scenario), 3 for soil, and 4 for sediment.

|  | ID | X (ESPG: 32632) | Y (ESPG: 32632) | type | data source | code |
| --- | --- | --- | --- | --- | --- | --- |
| Crocette | Cr 1 | 420752.4 | 5087951 | soil | previous | 3 |
|  | Cr 2 | 420723.4 | 5088010 | soil | previous | 3 |
|  | Cr 3 | 420738.4 | 5087918 | soil | previous | 3 |
|  | Cr 4 | 420720.4 | 5087919 | tailings | previous | 2 |
|  | Cr 6 | 420734.4 | 5087882 | soil | previous | 3 |
|  | Cr 7 | 420688.4 | 5087866 | tailings | previous | 1 |
|  | CRO 1 | 420656.4 | 5087868 | waste rock | previous | 2 |
|  | CRO 2 | 420686.4 | 5087859 | tailings | previous | 1 |
|  | CRO 3 | 420727.4 | 5087882 | soil | previous | 3 |
|  | CRO 4 | 420722.4 | 5087913 | tailings | previous | 2 |
|  | CRO 5 | 420757.4 | 5087873 | drainage channel sediments | previous | 4 |
|  | cr_01 | 420650.3 | 5087866 | waste rocks | new | 2 |
|  | cr_02 | 420650.3 | 5087866 | waste rocks | new | 2 |
|  | cr_03 | 420655.9 | 5087867 | waste rocks | new | 2 |
|  | cr_04 | 420655.9 | 5087867 | waste rocks | new | 2 |
|  | cr_05 | 420655.9 | 5087867 | waste rocks | new | 2 |
|  | cr_06 | 420672.8 | 5087848 | soil | new | 3 |
|  | cr_07 | 420707.6 | 5087866 | tailings | new | 1 |
|  | cr_08 | 420707.6 | 5087866 | tailings | new | 1 |
|  | cr_09 | 420705.2 | 5087913 | waste rocks | new | 2 |
|  | cr_10 | 420705.2 | 5087913 | tailings | new | 2 |
|  | cr_11 | 420731.0 | 5087911 | tailings | new | 2 |
|  | cr_12 | 420767.7 | 5087829 | soil | new | 3 |
|  | cr_13 | 420718.2 | 5087929 | tailings | new | 2 |
|  | cr_14 | 420686.9 | 5087863 | tailings | new | 1 |
| Pestarena | F 1 | 423732.4 | 5089876 | tailings | previous | 1 |
|  | F 3 | 423729.4 | 5089874 | tailings | previous | 1 |
|  | LM | 423737.4 | 5089865 | river sediments | previous | 4 |
|  | PES 12 | 423766.4 | 5089902 | drainage channel sediments | previous | 4 |
|  | PES 13 | 423769.4 | 5089901 | drainage channel sediments | previous | 4 |
|  | PES 14 | 423778.4 | 5089896 | river sediments | previous | 4 |
|  | Pst 1d | 423582.4 | 5089874 | waste rock | previous | 1 |
|  | Pst 1i | 423715.4 | 5089874 | tailings | previous | 1 |
|  | Pst 1v | 423982.4 | 5090035 | tailings | previous | 2 |
|  | Pst 2d | 423580.4 | 5089873 | waste rock | previous | 1 |
|  | Pst 2i | 423711.4 | 5089869 | tailings | previous | 2 |
|  | Pst 2v | 423983.4 | 5090033 | tailings | previous | 2 |
|  | Pst 4d | 423554.4 | 5089910 | soil | previous | 3 |
|  | Pst 4i | 423752.4 | 5089889 | tailings | previous | 1 |
|  | Pst 5i | 423733.4 | 5089894 | tailings | previous | 2 |
|  | Pst 6i | 423713.4 | 5089861 | soil | previous | 3 |
|  | Pst 7i | 423769.4 | 5089901 | drainage channel sediments | previous | 4 |
|  | S1 v | 423910.3 | 5089974 | tailings | previous | 1 |
|  | S10 | 424030.4 | 5090058 | soil | previous | 3 |
|  | S11 | 424034.4 | 5090043 | soil | previous | 3 |
|  | S12 | 424040.4 | 5090053 | tailings | previous | 2 |
|  | S14 | 423933.4 | 5089993 | tailings | previous | 2 |
|  | S15 | 423937.4 | 5089996 | tailings | previous | 2 |
|  | S16 | 423944.4 | 5089997 | tailings | previous | 2 |
|  | S17 | 423942.4 | 5090003 | tailings | previous | 2 |
|  | S18 | 423949.4 | 5090004 | tailings | previous | 2 |
|  | S19 | 423955.4 | 5090011 | tailings | previous | 2 |
|  | S2 v | 423915.4 | 5089978 | tailings | previous | 1 |
|  | S21 | 423961.4 | 5090016 | tailings | previous | 2 |
|  | S22 | 423967.4 | 5090021 | tailings | previous | 2 |
|  | S23 | 423974.4 | 5090023 | tailings | previous | 2 |
|  | S24 | 423976.5 | 5090028 | tailings | previous | 2 |
|  | S25 | 423982.4 | 5090024 | tailings | previous | 2 |
|  | S26 | 423991.4 | 5090027 | tailings | previous | 2 |
|  | S27 | 423994.4 | 5090033 | tailings | previous | 1 |
|  | S28 | 423999.4 | 5090030 | tailings | previous | 1 |
|  | S29 | 423998.4 | 5090034 | tailings | previous | 1 |
|  | S3 v | 424034.4 | 5090049 | tailings | previous | 1 |
|  | S30 | 424007.4 | 5090033 | tailings | previous | 1 |
|  | S31 | 424011.4 | 5090041 | tailings | previous | 2 |
|  | S32 | 424016.4 | 5090048 | soil | previous | 3 |
|  | S33 | 423736.4 | 5089899 | tailings | previous | 2 |
|  | S34 | 423731.4 | 5089882 | tailings | previous | 1 |
|  | S35 | 423740.4 | 5089892 | tailings | previous | 2 |
|  | S36 | 423750.4 | 5089902 | tailings | previous | 2 |
|  | S37 p | 423592.4 | 5089900 | soil | previous | 3 |
|  | S38 p2 | 423740.4 | 5089884 | tailings | previous | 2 |
|  | S39 p3 | 423751.4 | 5089892 | tailings | previous | 2 |
|  | S4 v | 424036.4 | 5090055 | tailings | previous | 1 |
|  | S40 | 423655.4 | 5089892 | soil | previous | 3 |
|  | S41 | 423666.4 | 5089899 | soil | previous | 3 |
|  | S42 | 423676 | 5089887 | soil | previous | 3 |
|  | S43 | 423633.5 | 5089853 | soil | previous | 3 |
|  | S44 | 423648.1 | 5089859 | soil | previous | 3 |
|  | S45 | 423635.1 | 5089900 | soil | previous | 3 |
|  | S5 | 423900.4 | 5089966 | tailings | previous | 2 |
|  | S6 | 423910.3 | 5089981 | tailings | previous | 2 |
|  | S7 | 423927.4 | 5089979 | tailings | previous | 2 |
|  | S8 | 423928.4 | 5089987 | tailings | previous | 2 |
|  | pe_01 | 423747.8 | 5089891 | tailings | new | 1 |
|  | pe_02 | 423727.4 | 5089874 | tailings | new | 1 |
|  | pe_03 | 423978.3 | 5090016 | drainage channel sediments | new | 4 |
|  | pe_04 | 423994.7 | 5090032 | tailings | new | 1 |
|  | pe_05 | 423743.3 | 5089894 | tailings | new | 1 |
|  | pe_06 | 423741 | 5089890 | tailings | new | 1 |
|  | pe_07 | 423751.4 | 5089880 | tailings | new | 2 |
|  | pe_08 | 423764.5 | 5089885 | river sediments | new | 4 |
|  | pe_09 | 423732.4 | 5089880 | tailings | new | 1 |
|  | pe_10 | 423726.1 | 5089882 | tailings | new | 1 |
|  | pe_11 | 423603.3 | 5089885 | waste rock | new | 1 |
|  | pe_12 | 424003.9 | 5090030 | tailings | new | 1 |
|  | pe_13 | 424012.2 | 5090038 | tailings | new | 1 |
|  | pe_14 | 423619 | 5089868 | waste rock | new | 1 |

As a complementary check of samples classification, Principal Component Analysis (PCA) and hierarchical clustering on multi-element compositions were also performed for both sites (Figs. S1–S2). Because EW has progressively mixed with the surrounding soils, samples assigned to different field-based categories do not necessarily correspond to geochemically distinct end-members; a compositional grouping is therefore not expected to sharply separate them. Consistent with this, the resulting statistical clusters did not correspond to the field-based classification. All analyses were performed in R (Version 2025.05.1+513)

**Fig. S1** Principal Component Analysis (left) and hierarchical clustering (right) of Pestarena samples. PCA of log-transformed, unit-variance-scaled multi-element geochemical data for Pestarena samples. Points are coloured and shaped according to the field-based classification (Code: 1 for indicated EW, 2 for inferred EW, 3 for soil, and 4 for sediment.). Dashed ellipses represent 95% confidence regions for each field-based category. PC1 and PC2 explain 45.8% and 32.2% of total variance, respectively. Ward's hierarchical clustering (k = 4), plotted in principal component space. Point colour indicates the statistical cluster assignment; point shape indicates the field-based classification. Correspondence between statistical clusters and field-based categories was close to null (Adjusted Rand Index = -0.02).

**Fig. S2** Principal Component Analysis (left) and hierarchical clustering (right) of Crocette samples. PCA of log-transformed, unit-variance-scaled multi-element geochemical data for Pestarena samples. Points are coloured and shaped according to the field-based classification (Code: 1 for indicated EW, 2 for inferred EW, 3 for soil, and 4 for sediment.). Dashed ellipses represent 95% confidence regions for each field-based category. PC1 and PC2 explain 37.6% and 26.6% of total variance, respectively. Ward's hierarchical clustering (k = 4), plotted in principal component space. Point colour indicates the statistical cluster assignment; point shape indicates the field-based classification. Correspondence between statistical clusters and field-based categories was close to null (Adjusted Rand Index = -0.01).

S2. **Data comparability**

Data from the 2024 and previous sampling periods were compared separately for each metal. Descriptive statistics (sample size, mean, and median) were calculated, and data normality was assessed using the Shapiro–Wilk test when applicable. Differences between periods were evaluated using the Mann–Whitney U test and Welch’s *t*-test. To account for multiple comparisons, *p*-values from the Mann–Whitney tests were adjusted using the Benjamini–Hochberg procedure, with statistical significance set at *p* < 0.05. All analyses were performed in R (Version 2025.05.1+513).

Table S2 Statistical comparison of elemental concentrations between the 2024 and previous sampling periods. Sample size (N), mean and median concentrations are reported for each element and period. Statistical significance is indicated in bold.

| **Element** | **N**  **2024** | **N**  **Previous** | **Mean**  **2024** | **Median**  **2024** | **Mean**  **Previous** | **Median**  **Previous** | **p**  **value** |
| --- | --- | --- | --- | --- | --- | --- | --- |
| **As** | 26 | 72 | 11379.3 | 5835.0 | 20225.7 | 9540.0 | 1.1E-01 |
| **Cd** | 26 | 55 | 0.3 | 0.3 | 1.1 | 0.3 | 7.9E-01 |
| **Co** | 26 | 55 | 30.8 | 27.5 | 9.8 | 4.8 | **5.8E-08** |
| **Cr** | 26 | 55 | 31.3 | 31.5 | 11.0 | 8.4 | **1.7E-05** |
| **Cu** | 26 | 55 | 22.3 | 15.5 | 56.4 | 31.3 | 9.7E-02 |
| **Ni** | 26 | 55 | 12.0 | 8.5 | 12.0 | 7.7 | 3.8E-01 |
| **Pb** | 26 | 55 | 299.9 | 118.5 | 600.7 | 270.0 | 9.7E-02 |
| **Sb** | 26 | 42 | 7.0 | 2.2 | 9.9 | 5.9 | 1.3E-01 |
| **V** | 26 | 42 | 47.2 | 43.5 | 13.7 | 10.0 | **4.6E-06** |
| **Zn** | 26 | 55 | 43.1 | 38.5 | 99.4 | 37.1 | 7.9E-01 |

S3. **Mineralogical analyses**

SEM-EDS analyses revealed (Fig. S1): (a) Monazite-(Ce), in granules ranging in size from approximately 60 to 180 µm. In some cases, traces of (b) ilmenite. Traces of residual (c) arsenopyrite and (d) pyrite. Traces of (e) zircon and (f) barite.

**Fig. S3** SEM-EDS images of: (a) Monazite-(Ce); (b) ilmenite; (c) arsenopyrite; (d) pyrite; (e) zircon; (f) barite

S4. **Environmental impacts on sediment samples**

| ID | Igeo | | | | | | | ERI | | | | | | | PERI | |  |
| --- | --- | --- | --- | --- | --- | --- | --- | --- | --- | --- | --- | --- | --- | --- | --- | --- | --- |
|  | As | Co | Cr | Cu | Ni | Pb | Zn | As | Co | Cr | Cu | Ni | Pb | Zn | |  | |
| LM | 0.8 | 1.3 | 0.1 | 1.2 | 0.2 | 0.1 | 0.3 | -4.2 | -2.6 | -4.8 | -2.7 | -5.5 | -6.5 | -2.3 | | 3.9 | |
| PES 12 | 439.8 | 17.9 | 0.9 | 25.5 | 4.2 | 33.3 | 4.2 | 4.9 | 1.3 | -1.7 | -1.7 | -0.8 | 2.1 | 1.5 | | 525.7 | |
| PES 13 | 492.1 | 18.1 | 0.9 | 30.0 | 4.9 | 35.4 | 4.8 | 5.0 | 1.3 | -1.7 | -1.7 | -0.6 | 2.2 | 1.7 | | 586.1 | |
| PES 14 | 2.1 | 1.9 | 0.2 | 3.6 | 1.1 | 0.5 | 0.2 | -2.9 | -2.0 | -3.7 | -3.7 | -2.8 | -4.0 | -2.7 | | 9.6 | |
| CRO 5 | 215.1 | 1.4 | 0.6 | 4.4 | 1.2 | 8.1 | 0.8 | 3.8 | -2.5 | -2.3 | -2.3 | -2.6 | 0.1 | -1.0 | | 231.5 | |
| Pst 7i | 263.5 | N.A | N.A | N.A | N.A | N.A | N.A | 4.1 | N.A | N.A | N.A | N.A | N.A | N.A | | 263.5 | |
| pe_03 | 59.5 | 8.5 | 1.1 | 3.7 | 2.2 | 4.5 | 0.6 | 2.0 | 0.2 | -1.5 | -1.5 | -1.8 | -0.7 | -1.4 | | 80.1 | |
| pe_08 | 1.3 | 10.6 | 0.8 | 1.6 | 1.6 | 1.4 | 0.5 | -3.5 | 0.5 | -1.9 | -1.9 | -2.3 | -2.4 | -1.7 | | 17.8 | |

S5. **Assessment of potentially recoverable CRM**

To make a preliminary assessment of the potential for CRM recovery, the concentrations detected in the analysed samples were compared with values reported in studies on secondary resources such as EW. As they depend on site-specific factors, these reference values are not absolute thresholds, nevertheless they provide a useful benchmark. The focus was on As, Be, Bi, Sb, W and REE, which showed enrichment (based on the normalised concentration to the UCC). Table S2 summarises the relevant literature data discussed above.

For Be, Lohmeier et al. (2021) studied tailings from a historic Sn mine and found an average concentration of approximately 140 mg/kg of Be. While there is no dedicated Be processing for recovery, the study proposes recovering Li alongside Be through gravimetric separation and flotation, which could make extracting around 1,600 tonnes of BeO economically viable. Li et al. (2025) studied Be-containing sludge residues from the neutralisation of wastewater from a Be smelting plant. They developed a method for extracting Be from an initial concentration of 0.14%, achieving an extraction efficiency of 95.82% under optimised conditions.

Han et al. (2021) points out that, at present, W tailings are currently still mainly treated as waste rather than potential resources, and that there is experiences of reprocessing. One early study was conducted by Clemente et al. (1993) at a W mine, where the recovery of wolframite from historical and recent tailings was investigated. The most promising approach was a combination of three-stage gravimetric separation and intermediate sulfide flotation. This resulted in concentrates containing 50–55% WO₃, with a recovery that seemed reasonable. More recently, Han et al. (2025) conducted reprocessing tests on EW characterised by an initial WO₃ content of 0.06%. They demonstrated the effectiveness of gravimetric separation and bioleaching for W recovery and environmental remediation. With the same objective Figueiredo et al. (2019) analysed a tailings dam with the same objective as part of the REMinE (Improve Resource Efficiency and Minimize Environmental Footprint) project. An average W concentration of 0.17% (up to 0.57% in deeper areas) was detected, as well as an As content of 13.55%. Although no recovery tests were conducted, the study proposes a treatment circuit based on gravimetric separation, which was considered technically feasible.

With regard to As, Bi and Sb: Choi et al. (2016) treated contaminated soil from an industrial area, which had an initial average As content of 332.47 mg/kg. Through oil agglomeration flotation, the arsenic content was reduced below the regulatory threshold, achieving a recovery efficiency of over 97%. Lemos et al. (2023) treated Au tailings containing 0.776% As and 0.08% Sb, enabling the recovery in the form of glass that could potentially be reused in industry. Radu et al. (2024) analysed tailings and WR from several historical mines. They found Bi concentrations of up to 180 mg/kg and Sb concentrations of up to 4534 mg/kg. While the study did not include recovery tests, it emphasised the consistent presence of these elements in mining waste that could be utilised as a valuable resource. Khmelnitskaya and Beskrovnaya (2007) analysed cyanidation tailings and leach residues from Au-Bi deposit with an initial content of 0.08% Bi. The recovery rate was between 75.8% and 76.9% when they were treated using an optimised acid leaching process.

Finally, a large number of studies on the recovery of REE from different secondary resources have been conducted all over the world. For example, Corchado-Albelo and Alagha (2023) analysed residues from Fe oxide processing in U.S. Reynier et al. (2021) studied tailings from U mines in Canada. Peelman et al. (2016) investigated residues from an Fe mine in Sweden. Fleming et al. (2021) analysed residues from Cu leaching processes in Chile. Abaka-Wood et al. (2022) studied tailings from Cu flotation in Australia. Echeverry-Vargas and Ocampo-Carmona (2022) analysed tailings from alluvial Au mining in Colombia. Together, these studies confirm the high potential for the recovery of REE from EW.

Table S4 Summary of literature data on the recovery of CRMs from secondary resources, including EW, contaminated soils, and industrial residues, reporting initial concentrations and main recovery outcomes.

| CRM | references | Secondary resource type | Initial concentration | Main results |
| --- | --- | --- | --- | --- |
| Be | (Lohmeier et al. 2021) | Tailings from historic Sn mine | 140.0 mg/kg Be | Potential extraction of 1600 t BeO |
|  | (Li et al. 2025) | Sludge from neutralisation of wastewater (Be smelting plant) | 0.1% Be | Extraction efficiency 95.82% |
| W | (Clemente et al. 1993) | W mine tailings | – | Concentrates with 50–55% WO₃, reasonable recovery |
|  | (Han et al. 2025) | Extractive waste | 0.1% WO₃ | W recovery |
| W/As | (Figueiredo et al. 2019) | W tailings dam | 0.8 % W, 13.5% As | Considered technically feasible |
| As | (Choi et al. 2016) | Contaminated soil (industrial area) | 332.47 mg/kg As | >97% Recovery efficiency |
| As/Sb | (Lemos et al. 2023) | Au tailings | 0.8% As, 0.08% Sb | Recovery as glass product |
| Sb/Bi | (Radu et al. 2024) | Tailings and WR from polymetallic historical mines | Up to 180.0 mg/kg Bi, 4534.0 mg/kg Sb | Occurrence of Bi and Sb as potential resources |
| Bi | (Khmelnitskaya and Beskrovnaya 2007) | Cyanidation tailings and leach residues from Au-Bi deposit | 0.1% Bi | Bi recovery 75.8–76.9% |
| REE | (Abaka-Wood et al. 2022) | Cu mine tailings | 4375.0 ppm TREE | Froth Flotation: >90% REE recovery; Magnetic Separation: up to 86%; Gravity separation (tabling): up to 54% |
|  | (Peelman et al. 2016) | Fe mine tailings | 5000.0 ppm TREE | HREE recovery 75–100% |
|  | (Reynier et al. 2021) | U mine tailings | 1330.0 ppm TREE | Recovery: LREE 81%, HREE 65% |
|  | (Echeverry-Vargas and Ocampo-Carmona 2022) | Alluvial Au tailings | Monazite concentrate: Ce 21.3 wt%, La 9.7 wt%, Nd 8.7 wt% | 100% extraction yield for La, Ce, Nd |
|  | (Corchado-Albelo and Alagha 2023) | Fe oxide processing residues | 11 000.0 ppm TREE | P₂O₅ recovery and indirect enrichment of REE |
|  | (Fleming et al. 2021) | Cu leaching residues | 579.8 ppm TREE | Max efficiency 64.5% total REE |

Examples of the concurrent recovery of As Sb, Bi, and W from different types of mining and processing residues have been reported in the literature. The most important method of producing As is as a by-product of other metallurgical operations (European Commission 2020; USGS 2021). Concurrently, elements such as Be, Bi, Sb, W and REEs occur at concentrations lower than those typically considered economically viable, even in secondary deposits. Nevertheless, their presence in EW is significant and provides opportunities for their potential recovery as associated CRMs. In pyrometallurgical Sb processing, the As contained in the ores is removed during oxidation, volatilisation and reduction smelting. Crude Sb is then refined using either sodium carbonate or sodium hydroxide, which removes the As and produces arsenic-alkali residues (AAR) (Guo et al. 2014; Dembele et al. 2022). AAR can be treated to recover As via thermal reduction. For this process, sodium arsenate is heated with charcoal powder under optimised conditions (800 °C for 60 minutes with 12% charcoal), resulting in the volatilisation of up to 99.84% of As in its elemental form (As⁰) (Yang et al. 2018). Another process involved two-step alkali recovery using CO₂, followed by deep alkali removal with H₂SO₄, reduction of As(V) with SO₂, and subsequent evaporation and concentration achieving As recovery of 80.6% as As₂O (Long et al. 2020).

From a mineral processing perspective, Sb ore beneficiation typically involves crushing, grinding, classification and flotation. This generates tailings containing stibnite, pyrite and minor amounts of arsenopyrite, as well as gangue minerals such as quartz and muscovite (Multani et al. 2016). Stibnite flotation tailings can be processed using hydrometallurgical methods involving acidic leaching with HCl and NaNO₃ as oxidising agents to selectively dissolve Sb and As (Dembele et al. 2024). Optimised conditions (4.4 M HCl, 0.5 M NaNO₃, 25% pulp density, 70 °C and 1 h leaching) achieve high extraction efficiencies of 98.3% for Sb and 92% for As.

Cu processing waste, such as anode slimes and flue dust, contains As, Sb and Bi reflecting their common occurrence in Cu ores (Dupont et al. 2016). However, Cu produced via pyrometallurgical processes often requires electrorefining, as As, Sb, and Bi negatively affect product quality. Ion exchange (IX) using amino-phosphonic resins (AMPIX) is widely applied industrially for the selective removal of impurities (Navarro and Alguacil 2002).

The effluent generated during the regeneration of AMPIX resins can be processed using selective precipitation to recover Sb and Bi. However, prior reduction of As(V) is necessary to prevent the formation of SbAsO₄ and BiAsO₄. Reducing agents such as SO₂ or NaHSO₃ effectively convert As(V) to As(III), which remains in solution. The eluate is then treated adding Fe₂(SO₄)₃, which stabilises the As as scorodite. Sb and Bi are then selectively precipitated through neutralisation with NaOH (5 M), producing Sb oxychloride (Sb₄O₅Cl₂), and Bi oxychloride (BiOCl) (Luo et al. 2023).

As-rich sludge from flue gas purification in Cu smelting can also be treated via desulfurisation–leaching–hydrolysis processes. First, the solid waste is desulfurised at 95 °C with kerosene. Then, chloride leaching is performed with H₂SO₄ and NaCl. Finally, controlled pH hydrolysis is used to precipitate Bi and Sb as oxychlorides (BiOCl and SbOCl) (Zhang et al. 2012).

Cu smelter electrostatic precipitator dust is processed through pilot-scale continuous hydrometallurgical leaching and selective precipitation to recover 99% of the Bi as a 68–77% pure oxychloride (BiOCl) precipitate, while concurrently generating Pb concentrates for secondary smelting and stabilized As fractions (Córdoba et al. 2026).

Directly from arsenic-rich Cu smelter dust As and Sb can be recovered by leaching with HCl solution (4.M, 363 K, 2 hours) to dissolve As₂O₃ and Sb₂O₃. This is followed by the reduction of As³⁺ to elemental As using sodium hypophosphite (NaH₂PO₂), and the subsequent recovery of Sb as SbCl₃ by continuous distillation. This approach achieves As recovery of up to 92.5% and SbCl₃ purity of over 97.5% (Xue et al. 2021).

Mining residues from W-bearing ores can be processed to recover W and As. Deep eutectic solvents, such as choline chloride: oxalic acid (ChCl:OA) when combined with electrodialysis (ED), can be used to treat Sn-W mining residues. Using a two-compartment ED setup with ChCl:OA (100 mA, 4 days), As and W were extracted from the residues, with up to 82% of As and 77% of W successfully migrating into the anode compartment for further separation (Almeida et al. 2020).

Scheelite can be processed to remove As and extract W through the formation and selective decomposition of arsenotungstic acid. The ore is leached with an H₃AsO₄–H₂SO₄ solution to form arsenotungstic acid, achieving a 99.9% W leaching rate. This acid is then selectively extracted using dodecanol and the resulting organic phase can be stripped with hot water to produce a pure solution. This solution reacts with ammonium to form ammonium arsenotungstate (AAT). Reducing AAT with hydrogen allows the As to volatilise and condense into elemental As, while the W is recovered as an ultrafine powder (Cao et al. 2024).

Tungsten residue waste (TRW) can be treated via a hydrometallurgical process involving alkaline pressure oxidation leaching. In TRW, As primarily occurs as arsenopyrite (FeAsS), co-precipitated with Mn(II) oxides and encapsulated in Fe–Mn oxides, while W is uniformly distributed within the same matrices. Leaching under optimized conditions (NaOH 60–90 g/L, 140–200 °C, 0.5–0.9 MPa O₂) enables selective dissolution of As and W, achieving efficiencies of approximately 97% and 82%, respectively. The leach solution is then subjected to solvent extraction with Alamine 336 to effectively separate W and As. W is almost completely recovered as pure sodium tungstate, while As remains in the raffinate, is reduced with SO₂, and finally precipitated as As₂S₃ (Wu et al. 2023).

**References**

Abaka-Wood GB, Addai-Mensah Jonas, and Skinner W (2022) The Use of Mining Tailings as Analog of Rare Earth Elements Resources: Part 1 – Characterization and Preliminary Separation. Miner Process Extr Metall Rev 43:701–715. https://doi.org/10.1080/08827508.2021.1920410

Almeida J, Craveiro R, Faria P, et al (2020) Electrodialytic removal of tungsten and arsenic from secondary mine resources — Deep eutectic solvents enhancement. Sci Total Environ 710:136364. https://doi.org/10.1016/j.scitotenv.2019.136364

Cao C, Fu A, Mai Q, et al (2024) Study on the integrated technology of arsenic removal and tungsten extraction based on the formation and decomposition of arsenotungstic acid. Chem Eng J 499:156963. https://doi.org/10.1016/j.cej.2024.156963

Choi J, Lee E, Choi SQ, et al (2016) Arsenic removal from contaminated soils for recycling via oil agglomerate flotation. Chem Eng J 285:207–217. https://doi.org/10.1016/j.cej.2015.09.105

Clemente D, Newling P, Botelho de Sousa A, et al (1993) Reprocessing slimes tailings from a tungsten mine. Miner Eng 6:831–839. https://doi.org/10.1016/0892-6875(93)90057-T

Corchado-Albelo JL, Alagha L (2023) Studies on the Enrichment Feasibility of Rare Earth-Bearing Minerals in Mine Tailings. Minerals 13:301. https://doi.org/10.3390/min13030301

Córdoba P, Mayor L, Espí JJ, Licon E (2026) Addressing sustainability trade-offs in critical raw material recovery from metallurgical residues: An integrated environmental–economic–strategic framework. Clean Eng Technol 32:101226. https://doi.org/10.1016/j.clet.2026.101226

Dembele S, Akcil A, Panda S (2022) Technological trends, emerging applications and metallurgical strategies in antimony recovery from stibnite. Miner Eng 175:107304. https://doi.org/10.1016/j.mineng.2021.107304

Dembele S, Akcil A, Panda S (2024) Investigation of the characteristics of stibnite (Sb2S3) flotation tailings and extraction of critical metals (Sb and As): Optimization and scale-up. Miner Eng 216:108883. https://doi.org/10.1016/j.mineng.2024.108883

Dupont D, Arnout S, Jones PT, Binnemans K (2016) Antimony Recovery from End-of-Life Products and Industrial Process Residues: A Critical Review. J Sustain Metall 2:79–103. https://doi.org/10.1007/s40831-016-0043-y

Echeverry-Vargas L, Ocampo-Carmona LM (2022) Recovery of Rare Earth Elements from Mining Tailings: A Case Study for Generating Wealth from Waste. Minerals 12:. https://doi.org/10.3390/min12080948

European Commission: Directorate-General for Internal Market I Entrepreneurship and SMEs, Blengini GA, El Latunussa C, et al (2020) Study on the EU’s list of critical raw materials (2020) – Final report. Publications Office of the European Union

Figueiredo J, Vila MC, Góis J, et al (2019) Bi-level depth assessment of an abandoned tailings dam aiming its reprocessing for recovery of valuable metals. Miner Eng 133:1–9. https://doi.org/10.1016/j.mineng.2018.12.016

Fleming P, Orrego P, Pinilla F (2021) Recovery of Rare Earth Elements Present in Mining Tails, by Leaching with Nitric and Hydrochloric Solutions. World J Nucl Sci Technol 11:1–16. https://doi.org/10.4236/wjnst.2021.111001

Guo X, Wang K, He M, et al (2014) Antimony smelting process generating solid wastes and dust: Characterization and leaching behaviors. J Environ Sci 26:1549–1556. https://doi.org/10.1016/j.jes.2014.05.022

Han Z, Edraki M, Golev A (2025) Characterization, potential valuable metals recovery and remediation of historic wolframite mining waste. Sep Purif Technol 354:129009. https://doi.org/10.1016/j.seppur.2024.129009

Han Z, Golev A, Edraki M (2021) A Review of Tungsten Resources and Potential Extraction from Mine Waste. Minerals 11:701. https://doi.org/10.3390/min11070701

Khmelnitskaya OD, Beskrovnaya VP (2007) A technology of precious metals recovery from gold-bismuth ores. In: Australas. Inst. Min. Metall. Publ. Ser. pp 245–250

Lemos MG, Valente TM, Marinho Reis AP, et al (2023) Adding Value to Mine Waste through Recovery Au, Sb, and As: The Case of Auriferous Tailings in the Iron Quadrangle, Brazil. Minerals 13:. https://doi.org/10.3390/min13070863

Li L, Gao H, Li J, et al (2025) A new insight into beryllium extraction mechanism from beryllium-containing sludge through key host phase reconstruction and binding energy regulation. J Clean Prod 489:144667. https://doi.org/10.1016/j.jclepro.2025.144667

Lohmeier S, Lottermoser BG, Schirmer T, Fuchsloch W (2021) Reprocessing potential of pegmatite tailings for rare metal extraction and brick fabrication, Uis, Namibia. South Afr J Geol 124:639–662. https://doi.org/10.25131/sajg.124.0015

Long H, Zheng Y, Peng Y, et al (2020) Separation and recovery of arsenic and alkali products during the treatment of antimony smelting residues. Miner Eng 153:106379. https://doi.org/10.1016/j.mineng.2020.106379

Luo D, Fernández de Labastida M, Cortina JL, Lopez J (2023) Recovery of antimony and bismuth from arsenic-containing waste streams from the copper electrorefining circuit: An example of promoting critical metals circularity from secondary resources. J Clean Prod 415:137902. https://doi.org/10.1016/j.jclepro.2023.137902

Multani RS, Feldmann T, Demopoulos GP (2016) Antimony in the metallurgical industry: A review of its chemistry and environmental stabilization options. Hydrometallurgy 164:141–153. https://doi.org/10.1016/j.hydromet.2016.06.014

Navarro P, Alguacil FJ (2002) Adsorption of antimony and arsenic from a copper electrorefining solution onto activated carbon. Hydrometallurgy 66:101–105. https://doi.org/10.1016/S0304-386X(02)00108-1

Peelman S, Sun Z h. i., Sietsma J, Yang Y (2016) Hydrometallurgical Extraction of Rare Earth Elements From Low Grade Mine Tailings. In: Rare Metal Technology 2016. John Wiley & Sons, Ltd, pp 17–29

Radu V-M, Dinca G, Ivanov A-A, et al (2024) New data regarding the identification of critical raw materials recoverable from raw, processed and the waste mining industry materials from Romania. Environ Sci Pollut Res 31:40592–40608. https://doi.org/10.1007/s11356-023-26536-x

Reynier N, Gagné-Turcotte R, Coudert L, et al (2021) Bioleaching of Uranium Tailings as Secondary Sources for Rare Earth Elements Production. Minerals 11:302. https://doi.org/10.3390/min11030302

USGS (2021) Mineral commodity summaries 2021. Reston, VA

Wu X, Qing J, Li J, et al (2023) A green process for arsenic removal and tungsten recovery from tungsten residue waste: Focused on the separation and recovery of arsenic and tungsten via solvent extraction followed by H2S precipitation. J Environ Chem Eng 11:110952. https://doi.org/10.1016/j.jece.2023.110952

Xue J, Long D, Zhong H, et al (2021) Comprehensive recovery of arsenic and antimony from arsenic-rich copper smelter dust. J Hazard Mater 413:125365. https://doi.org/10.1016/j.jhazmat.2021.125365

Yang K, Qin W, Liu W (2018) Extraction of Metal Arsenic from Waste Sodium Arsenate by Roasting with Charcoal Powder. Metals 8:542. https://doi.org/10.3390/met8070542

Zhang B, Li Q, Shen W, Min X (2012) Recovery of bismuth and antimony metals from pressure-leaching slag. Rare Met 31:102–106. https://doi.org/10.1007/s12598-012-0471-1
